# Bioactive glasses incorporating less-common ions to improve biological and physical properties

**DOI:** 10.1007/s10856-021-06626-3

**Published:** 2021-12-23

**Authors:** Usanee Pantulap, Marcela Arango-Ospina, Aldo R. Boccaccini

**Affiliations:** Department of Materials Science and Engineering, Institute of Biomaterials, University of Erlangen-Nuremberg, 91058 Erlangen, Germany

## Abstract

Bioactive glasses (BGs) have been a focus of research for over five decades for several biomedical applications. Although their use in bone substitution and bone tissue regeneration has gained important attention, recent developments have also seen the expansion of BG applications to the field of soft tissue engineering. Hard and soft tissue repair therapies can benefit from the biological activity of metallic ions released from BGs. These metallic ions are incorporated in the BG network not only for their biological therapeutic effects but also in many cases for influencing the structure and processability of the glass and to impart extra functional properties. The “classical” elements in silicate BG compositions are silicon (Si), phosphorous (P), calcium (Ca), sodium (Na), and potassium (K). In addition, other well-recognized biologically active ions have been incorporated in BGs to provide osteogenic, angiogenic, anti-inflammatory, and antibacterial effects such as zinc (Zn), magnesium (Mg), silver (Ag), strontium (Sr), gallium (Ga), fluorine (F), iron (Fe), cobalt (Co), boron (B), lithium (Li), titanium (Ti), and copper (Cu). More recently, rare earth and other elements considered less common or, some of them, even “exotic” for biomedical applications, have found room as doping elements in BGs to enhance their biological and physical properties. For example, barium (Ba), bismuth (Bi), chlorine (Cl), chromium (Cr), dysprosium (Dy), europium (Eu), gadolinium (Gd), ytterbium (Yb), thulium (Tm), germanium (Ge), gold (Au), holmium (Ho), iodine (I), lanthanum (La), manganese (Mn), molybdenum (Mo), nickel (Ni), niobium (Nb), nitrogen (N), palladium (Pd), rubidium (Rb), samarium (Sm), selenium (Se), tantalum (Ta), tellurium (Te), terbium (Tb), erbium (Er), tin (Sn), tungsten (W), vanadium (V), yttrium (Y) as well as zirconium (Zr) have been included in BGs. These ions have been found to be particularly interesting for enhancing the biological performance of doped BGs in novel compositions for tissue repair (both hard and soft tissue) and for providing, in some cases, extra functionalities to the BG, for example fluorescence, luminescence, radiation shielding, anti-inflammatory, and antibacterial properties. This review summarizes the influence of incorporating such less-common elements in BGs with focus on tissue engineering applications, usually exploiting the bioactivity of the BG in combination with other functional properties imparted by the presence of the added elements.

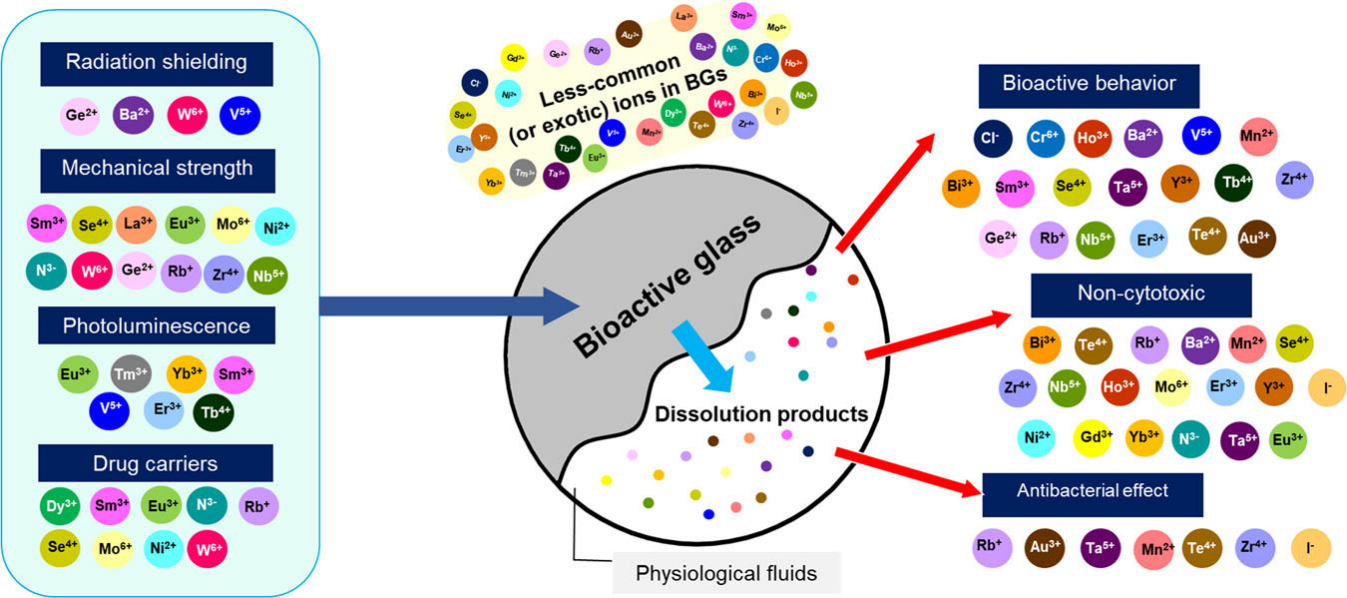

## Introduction

Bioactive glasses (BGs) are being increasingly investigated for both bone and soft tissue engineering applications [1, 2]. BGs exhibit a unique bone-bonding ability by forming a hydroxyapatite surface layer after incubation in physiological fluids and simultaneously support biological regenerative processes such as angiogenesis and osteogenesis during their dissolution [3, 4]. Furthermore, specific compositions of BGs can provide antibacterial activity [5–8] and/or induce an anti-inflammatory response [9, 10]. BGs have thus great potential in bone regeneration, drug delivery systems, as well as in soft tissue repair and wound healing [11, 12]. In 1969, Hench et al. used the Na_2_O–CaO–SiO_2_ phase diagram to develop the first BG, named “45S5 BG,” with composition: 45 SiO_2_–24.5 CaO–6 P_2_O_5_–24.5 Na_2_O (in wt.%). 45S5 BG has been considered in medical applications since 1985. The first 45S5 BG surgical implants were solid parts used to replace the small bones in the middle ear to treat conductive hearing loss [13]. Over the last 50 years, numerous BG compositions in the silicate, borosilicate, borate, and phosphate systems have been developed and characterized [14–16]. In general, the addition of glass modifiers has significant effects on glass properties, including bioactivity. BG compositions similar to 45S5 BG have been investigated. For example, ICIE16-BG [17], with a higher amount of CaO and lower amount of Na_2_O compared to 45S5 BG, along with K_2_O, has been shown to exhibit a larger sintering window that allows the shaping of 3D structures without crystallization [18, 19]. Another silicate BG that has received much attention is the 13–93 composition, which has shown less tendency to crystallize when sintered and is known to generate 3D scaffolds with superior mechanical properties [20, 21]. Moreover, boron-containing BGs have demonstrated that boron addition into silicate BGs enhances the degradation rate [16], the process of apatite formation [22, 23], antibacterial properties [23], osteogenesis [24–26], angiogenesis [26–28], and has also an effect on the BG mechanical strength [22, 29]. Boron-doped BGs have been shown to be attractive materials for applications in soft and hard tissue engineering [15, 30]. The chemical composition of phosphate-based BGs has also been studied to tailor the glass structure and to improve dissolution behavior and bioactive characteristics for biomedical applications [31, 32]. The modification of chemical compositions of BGs has been investigated as an approach to improve mechanical properties and glass durability. For example, aluminum ions have been incorporated in BGs to reinforce mechanical performance. Various studies have investigated Al_2_O_3_-doped 45S5 BGs (sol–gel and melt-derived) in terms of bioactivity and physical properties, demonstrating improved mechanical properties but reduced bioactivity for compositions with more than 1 mol% Al_2_O_3_ compared to bare 45S5 BGs. Moreover, sol–gel glasses with low amounts of Al_2_O_3_ (0.5–1 mol%) showed enhanced mechanical properties without significant reduction of bioactivity [33–36].

Biologically active ions have become widely used for enhancing the biological and physical effectiveness of BGs, aiming at developing multifunctional biomaterials for a wide range of biomedical applications. Metallic ions are not only essential for the human health but also could be an alternative to highly-priced pharmaceuticals [37, 38]. Significant research has been published on incorporating metallic ions (or bioinorganics) in BGs [39–43] as well as in the field of calcium phosphates [44–46]. The use of several biologically active ions has been prevalent in recent years, namely, Ag^+^, Li^+^, Co^2+^, Ca^2+^, Cu^2+^, Zn^2+^, Sr^2+^, Fe^2+^, Mg^2+^, Ga^3+^, and B^3+^ have been added to silicate, phosphate, and borate BG systems to promote functional properties such as osteogenesis, angiogenesis, bioactivity, antibacterial effects, and immunomodulation for tissue regeneration, as well as for infection and cancer treatment [40, 47, 48]. Several comprehensive reviews on such BGs incorporating “common” biologically active ions are available [8, 15, 31, 39–42, 49–54].

**Fig. 1:**
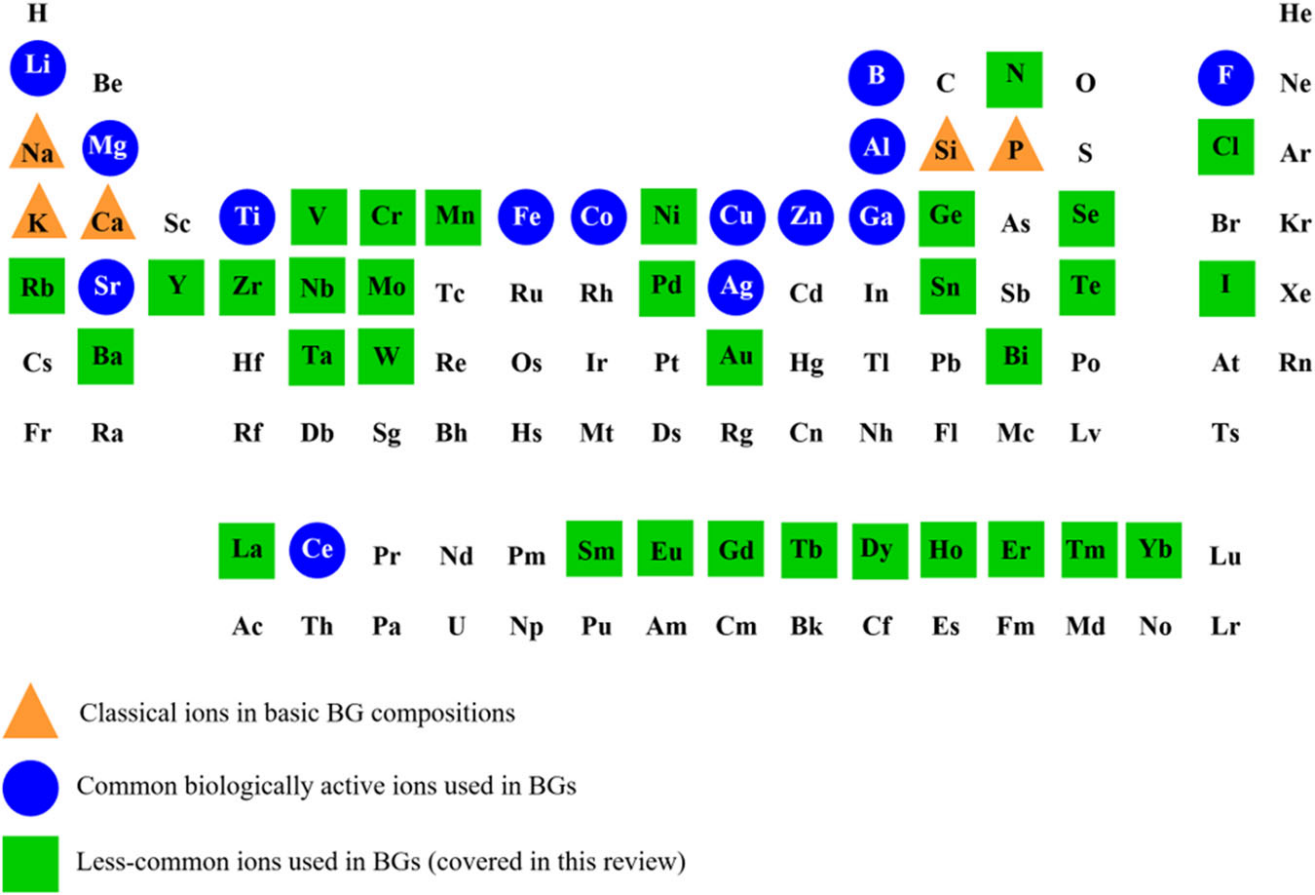
Periodic table of the elements highlighting the classical ions used to produce BGs, ions highly investigated to provide biological and therapeutic properties to BGs, and less-common ions in BGs, which are the ones covered in this review

**Fig. 2:**
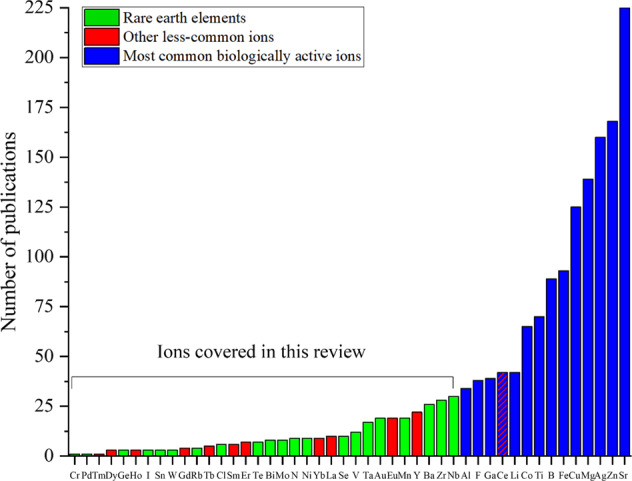
Number of publications in the last 20 years containing the keywords “bioactive glasses” or “bioglass” and the corresponding ions. The criteria used for the search considered that the keywords should appear on the title of the publication and//or the abstract. Data obtained from the database Scopus (www.scopus.com) and Web of Science (www.webofscience.com)

Recently, a significant number of BGs doped with what can be called less-common (or even exotic) ions, including rare earth elements, have started to be reported. Such BGs are attractive for tissue regeneration applications because of the functional properties, biological activity, and therapeutic effects provided by such ions. There has been no previous review article focusing on the development and applications of such BGs containing less-common ions. Therefore, this review article covers comprehensively literature reports on less-common ion-doped BGs, which include rare earth, metal, and non-metal elements: Ba^2+^, Bi^3+^, Cl^–^, Cr^6+^, Dy^3+^, Eu^3+^, Gd^3+^, Yb^3+^, Th^3+^, Ge^2+^, Au^3+^, Ho^3+^, I^–^, La^3+^, Mn^2+^, Mo^6+^, Ni^2+^, Nb^5+^, N^3^^–^, Pd^2+^, Rb^+^, Sm^3+^, Se^4+^, Ta^5+^, Te^4+^, Tb^3+^, Er^3+^, Sn^2+^, W^6+^, V^5+^, Y^3+^, and Zr^4+^. Figure 1 shows the periodic table of the elements highlighting the different ions that are considered basic constituents for the production of BGs or those mainly used to impart biological and therapeutic functionalities to BGs. An overview of BG formulations incorporating less-common ions, their applications and properties, including the synthesis method, is presented in Table 1 for rare earth elements and Table 2 for other less-common (biologically active) ions. Considering the increasing number of publications in the field of ion-doped BGs, the authors proposed a basic classification of the ions based on their primary function in the BG and, for the purpose of this review, the number of studies that have considered the respective ions for their biological effects. Based on the information shown in Fig. 2, the selection of ions for such classification, and thus the decision on which publications should be included in this review, was done considering the number of publications reporting the application of a given ion in BGs in the last 20 years. Ions used in less than 30 publications (up to August 31, 2021) were considered “less-common ions” and were thus included in this review (clearly this is an arbitrarily chosen number, but necessary to establish a criterion to identify such less-common ions).

**Table 1:** Compositions of rare earth elements-containing bioactive glasses for medical applications

Ion	Glass composition	Applications	Synthesis technique	Additional formation	Ref.
Dysprosium (Dy)	61.2 B_2_O_3_–8.8 Li_2_O–61.2 Dy_2_O_3_ (wt.%)	Drug delivery and radiation therapy	Melt-quenching	Microspheres with a particles size range from 45 to 150 µm	[133]
	50 SiO_2_–30 CaO–10 Fe_2_O_3_–10 Dy_2_O_3_ (mol%)	Radiotherapy and hyperthermia	Sol–gel	Porous glass powder after thermal treatment at 500 and 800 °C	[135]
Europium (Eu)	70 SiO_2_–20 CaO–5 P_2_O_5_ with 5 Eu_2_O_3_ (or Tb_2_O_3_) (mol%)	Bone regeneration and drug delivery	Sol–gel	Mesoporous nanofibers with an average diameter of 100–120 nm	[59]
	100 SiO_2_ with 1, 2, and 3 Eu_2_O_3_ (mol%)	Skin and bone regeneration	Sol–gel	Mesoporous nanospheres with a particle size range of 280-300 nm	[69]
	SiO_2_–CaO–P_2_O_5_ with 5 Eu_2_O_3_ (mol%)	Drug delivery	Sol–gel	Mesoporous powder	[58]
	60 SiO_2_–36 CaO–4 P_2_O_5_ with 0, 0.5, 1, and 2 Eu_2_O_3_ (mol%)	Bone regeneration	Sol–gel	Mesoporous nanospheres with a particle size around 500 nm	[57]
	80 SiO_2_–16 CaO–4 P_2_O_5_ with 1, 2, and 3 Eu_2_O_3_ (mol%)	Cell imaging and bone regeneration	Sol–gel	Nanoparticles with a particle size range of 200–400 nm	[60]
	80 SiO_2_–15 CaO–5 P_2_O_5_ with 0, 1, 2, and 5 Eu_2_O_3_ (mol%)	Cell imaging and bone regeneration	Sol–gel	Mesoporous bioactive glass scaffolds with a pore size range of 300–500 µm	[70]
Gadolinium (Gd), Ytterbium (Yb) and Thulium (Tm)	47.28 SiO_2_–31.39 Na_2_O–15.33 CaO–6 P_2_O_5_ with 2.5 Gd_2_O_3_ or Yb_2_O (wt.%)	Tissue engineering	Melt-quenching	Glass powder with a particle size of less than 125 µm	[85]
	SiO_2_–CaO–Gd_2_O_3_ with the Ca:Gd molar ratios 3:1 and 5:1	Bone regeneration	Sol–gel	Combination of mesoporous calcium silicate scaffold with chitosan using lyophilization technique	[90]
	84 SiO_2_–12 CaO–4 P_2_O_5_ with the Ca:Gd molar ration 3:1, 5:1, and 7:1	Bone regeneration	Sol–gel	Microsphere powder with a particle size around 300 nm + BG scaffold using lyophilization technique	[89]
	47.28 SiO_2_–31.39 NaO_2_–15.33 CaO–6 P_2_O_5_ with 2.5 Gd_2_O_3_ or 2.5 Yb_2_O_3_ or 0.5 Fe_2_O_3_ (wt.%)	Biomedical applications	Melt-quenching	Glass powder with a particle size of less than 75 µm	[84]
	63 SiO_2_–37 CaO with 0.15, 0.3 and 0.5 Tm_2_O_3_ and 0, 1, 2, 3 and 4 Yb_2_O_3_ (mol.%)	Regenerative medicine or drug delivery	Sol–gel	Glass powder with a particle size range of 80–120 nm	[91]
Holmium (Ho)	58 SiO _2_–33 CaO–9 P_2_O_5_ with 1.25, 2.5 and 5 Ho_2_O_3_ (wt.%)	Brachytherapy	Sol–gel	Glass powder	[78]
	58 SiO_2_–33 CaO–9 P_2_O_5_ with 1.25, 2.5, 3.75, and 5 Ho_2_O_3_ (wt.%)	Brachytherapy	Sol–gel	Glass powder incorporated into the Poloxamer 407 hydrogel (20 wt.%)	[79]
Lanthanum (La)	67 SiO_2_–5 Na_2_O–24 CaO–4 P_2_O_5_ with 5 La_2_O_3_ (or CuO) (mol%)	Tissue engineering	Sol–gel	Glass powder with a particle size of less than 63 µm and BG scaffolds with macropores in the range of 300–500 µm using the robocasting technique	[124]
	64.4 SiO_2_–2.48 Na_2_O–21.53 CaO–4.55 P_2_O_5_ with 0, 1, 3 and 5 wt.% La_2_O_3_ (or/and CuO)	Tissue engineering	Sol–gel	Glass powder with a particle size range of 3.5–4.6 µm	[123]
	25 Na_2_O–25 CaO–50 P_2_O_5_ with 5 and 10 La_2_O_3_ (mol%)	Drug delivery	Sol–gel	Mesoporous nanoparticles with a particle size range of 25–100 nm	[118]
	58 SiO_2_–38 CaO–4 P_2_O_5_–1 La_2_O_3_ (wt.%)	Bone regeneration	Sol–gel	Glass powder	[125]
	20 Na_2_O–14 CaO–66 P_2_O_5_ with 0, 0.1, 0.3, 0.7 and 1 La_2_O_3_ (mol%)	Tissue engineering	Melt-quenching	Glass powder with a particle size range of 106–180 µm	[116]
Samarium (Sm)	45 SiO_2_–24.5 Na_2_O–24.5 CaO–6 P_2_O_5_ with 0, 1, 2, 3, and 4 Sm_2_O_3_ (wt.%)	Bone regeneration	Melt-quenching	Glass powder	[97]
	46.1 SiO_2_–24.4 Na_2_O–26.9 CaO–6 P_2_O_5_ with 0, 0.2, and 2 Sm_2_O_3_ (wt.%)	Tissue engineering	Melt-quenching	Glass powder with a particle size of around 100 µm	[93]
	SiO_2_–CaO–P_2_O_5_ with 0, 0.5, and 1 Sm_2_O_3_ (mol%)	Bone cancer	Sol–gel	Combination of mesoporous bioactive glass with alginate powder with a particle size of around 1200 µm	[98]
	45.6 SiO_2_–24.4 Na_2_O–26.9 CaO–2.6 P_2_O_5_ with 0.5 Sm_2_O_3_ (mol%)	Biomedical applications	Melt-quenching	Glass fiber with a diameter of 100 µm from the glass melt	[96]
	10 Na_2_O–15 CaO–65 P_2_O_5_–15 CaF_2_ with 0, 0.5, 1, and 2 Sm_2_O_3_ (mol%)	Bone regeneration	Melt-quenching	Mixing of 2.5% glass powder with 97.5% of hydroxyapatite powder (wt.%)	[99]
Terbium (Tb) and Erbium (Er)	79.5 SiO_2_–15 CaO–5 P_2_O_5_ with 0.5 and 1 Tb_2_O_3_ (mol%)	Bone regeneration	Sol–gel	Mesoporous nanospheres with a particle size range of 100–200 nm	[130]
	53 SiO_2_–6 Na_2_O–20 CaO–4 P_2_O_5_–12 K_2_O–5 MgO with 1, 3, 5 Tb_2_O_3_ or 1, 3, 5 Er_2_O_3_ or 0.5, 1.5, and 2.5 with co-dopingTb_2_O_3_ and Er_2_O_3_ (wt.%)	Bioimaging	Sol–gel	Glass powder with a particle size range of 1.45–3.57 µm	[132]
	30 Na_2_O–25 CaO–45P_2_O_5_ with 0, 1, 3, and 5 Y_2_O_3_ (mol%)	Radiotherapy	Melt-quenching	Glass powder	[113]
	62.35 SiO_2_–15.85 Na_2_O–(20.80–*x*) CaO–1.0 P_2_O_5_ with *x* = 0 and 4.68 Y_2_O_3_ (mol%)	Radiotherapy	Melt-quenching	Glass powder	[112]
	58 SiO_2_–33 CaO–9 P_2_O_5_ with 10 Y_2_O_3_ (wt.%)	Radiotherapy	Sol–gel	Glass powder with an average particle size of 1 µm	[105]
Yttrium (Y)	6 Na_2_O–20 CaO–4 P_2_O_5_–12 K_2_O–5 MgO–52 B_2_O_3_–1 Y_2_O_3_ (wt.%)	Tissue engineering	Melt-quenching	Glass powder with a particle size range of 100–300 µm	[114]

**Table 2:** Formulations of bioactive glasses incorporating less-common elements according to the envisaged medical applications

Ion	Glass composition	Applications	Synthesis technique	Additional formation	Ref.
Barium (Ba)	44.85 SiO_2_–24.3 Na_2_O–26.9 CaO–2.6 P_2_O_5_–1.35 BaO (mol%)	Tissue engineering	Sol–gel	Glass powder with a particle size range of 508 ± 39 and 403 ± 42 nm	[9]
	60 SiO_2_–36 CaO–4 P_2_O_5_ with 0, 5, and 10 BaO and 0, 10, and 15 Fe_2_O (mol%)	Cancer hyperthermia	Sol–gel	Glass powder with a particle size range of 100–200 nm	[154]
	15 SiO_2_–20 Na_2_O–10 CaO–50 B_2_O_3_–5 Al_2_O_3_ with 0, 5, 10, 20, and 30 BaO (wt.%)	Radiation shielding	Melt-quenching	Glass powder	[160]
Bismuth (Bi)	53 SiO_2_–23 Na_2_O–20 CaO–4 P_2_O_5_ with 1, 2, 4, and 8 Bi_2_O_3_ (wt.%)	Bone regeneration	Melt-quenching	Glass powder with a particle size less than 45 µm	[346]
Chlorine (Cl)	50 SiO_2_–50 CaO with 0–43.1 CaCl_2_ (mol%)	Toothpaste additives	Melt-quenching	Glass	[325]
	38.1 SiO_2_–55.5 CaO–6.3 P_2_O_5_ with 0–16.6 CaCl_2_ (mol%)	Bone regeneration	Melt-quenching	Glass powder with a particle size less 38 μm	[327]
	38.1 SiO_2_–55.5 CaO–6.3 P_2_O_5_ with 0–21.5 CaCl_2_ and 0–13.4 CaF_2_ (mol%)	Dental toothpastes or resorbable bone substitutes	Melt-quenching	Glass powder with a particle size less 45 μm	[329]
Chromium (Cr)	5 SiO_2_–20 Na_2_O–20 CaO–2 P_2_O_5_–43 B_2_O_3_ with 0–1 Cr_2_O_3_ (mol%)	Bone regeneration	Melt-quenching	Glass powder	[241]
Germanium (Ge)	48 SiO_2_–12 CaO–36 ZnO with 0, 6.5, 7, and 8 GeO_2_ (mol%)	Bone filling materials	Melt-quenching	Glass powder with a particle size around 6 µm	[335]
	48 SiO_2_–6 CaO–2 P_2_O_5_–36 ZnO–8 SrO with 6 and 12 GeO_2_ (mol%)	Spinal orthopedic procedures	Melt-quenching	Glass powder with a maximum particle size of 45 μm	[336]
	9.9 Na_2_O–51. P_2_O_5_–20.8 K_2_O–8 BaO–7.2 Al_2_O_3_–0.2 Sb_2_O_3_–0.2188 La_2_O_3_–0.5 Nb_2_O_5_–0.5 Y_2_O_3_–0.9 Yb_2_O_3_ with 0.7–84.4 GeO_2_ (mol%)	Nuclear radiation shielding applications	Melt-quenching	Glass	[337]
Gold (Au)	60 SiO_2_–32 CaO–8 P_2_O_5_ with 0, 0.05, 0.075, 0.1, 0.15, and 0.2 Au_2_O (mol%)	Biomaterial	Sol–gel	Glass powder with a particle size about 100 µm	[305]
	60 SiO_2_–36 CaO–4 P_2_O_5_ (mol%) with 0.1 and 1 (wt%) gold nanoparticles	Biomaterial	Sol–gel	Glass powder	[306]
Iodine (I)	6 Na_2_O–20 CaO–4 P_2_O_5_–12 K_2_O–5 MgO–52.9 B_2_O_3_–0.1 I (wt.%)	Tissue engineering	Melt-quenching	Glass powder with a particle size range of 100–300 µm	[114]
	6 Na_2_O–20 CaO–4 P_2_O_5_–10 K_2_O–5 MgO–53 B_2_O_3_–2 I (wt.%)	Bone regeneration	Melt-quenching	Glass powder with a particle size less than 45 µm	[331]
	6 Na_2_O–20 CaO–4 P_2_O_5_–12 K_2_O–5 MgO–52 B_2_O_3_ (wt.%) with 0.2 wt.% NaI	Nerve regeneration	Melt-quenching	Glass powder (50 wt.%) incorporated into the PCL polymer (50 wt.%)	[332]
Manganese (Mn)	5 SiO_2_–20 Na_2_O–15 CaO–55 P_2_O_5_–5 B_2_O_3_ with 0, 0.1, 0.2, 0.3, 0.4, 0.5, 0.8, and 1 MnO (wt.%)	Bone regeneration	Melt-quenching	Glass powder	[288]
	60 SiO_2_–36 CaO–4 P_2_O_5_ with 0, 2.5, and 5 MnO_2_ (mol%)	Bone regeneration	Sol–gel	Glass powder with a particle size of less than 150 µm	[278]
	60 SiO_2_–36 CaO–4 P_2_O_5_ with 0, 1, 2.5, and 5 MnO (mol%)	Bone regeneration	Sol–gel	Glass powder with a particle size range of 38–150 µm	[284]
	43.29 SiO_2_–4.49 Na_2_O–31.02 CaO–11 P_2_O_5_–0.19 K_2_O–2.76 MgO–0.50 La_2_O_3_–0.99 Ta_2_O_5_–0.89 MnO (wt.%)	Coatings	Sol–gel	Glass powder	[388]
	50 SiO_2_–40 CaO–10 P_2_O_5_ with 0 and 5 MnO (mol%)	Bone regeneration	Sol–gel	Mesoporous powder with a particle size range of 100–120 nm	[287]
	45 Si_2_O–15 Na_2_O–26 CaO–3 P_2_O_5_–4 K_2_O–7 MgO with 0, 0.25, and 0.5 MnO (mol%)	Bone regeneration	Melt-quenching	Glass powder with a particle size of less than 32 µm	[282]
	50 SiO_2_–40 CaO–10 P_2_O_5_ with 0, 3, 5, and 7 MnO (mol%)	Bone regeneration	Sol–gel	Mesoporous powder with a particle size range of 110 ± 10 nm	[285]
	60 SiO_2_–36 CaO–4 P_2_O_5_ with 0, 3, and 5 MnO (mol%)	Bone regeneration	Sol–gel	Glass powder	[283]
	92 SiO_2_–8 CaO with 0, 3.3, and 4.2 MnO (mol%)	Tissue regeneration	Sol–gel	Glass powder with a particle size range of 112.2 ± 13.5 and 139.6 ± 8.9 nm	[389]
Molybdemiun (Mo)	70 SiO_2_–25 CaO–5 P_2_O_5_ with 0, 2, 5, and 7.5 MoO_3_ (mol%)	Cartilage/bone	Sol–gel	Scaffolds with cylindrical pores with an approximate diameter of 8 mm and height of 2 mm using 3D printing	[256]
	60 SiO_2_–30 CaO–10 P_2_O_5_ with 0, 3, 5, and 10 MoO_3_ (mol%)	Interface regeneration	Sol–gel	Glass powder	[255]
	45 CaO–48 P_2_O_5_–5 K_2_O–2 B_2_O_3_ with 0, 1, 3, 5, and 7 MoO_3_ (mol%)	Bone regeneration	Melt-quenching	Glass powder	[257]
Nickel (Ni)	46.1 SiO_2_–24.5 Na_2_O–26.9 CaO–2.6 P_2_O_5_ with 0, 0.41, 0.82, 1.23, and 1.65 Nb_2_O_5_ (mol%)	Bone regeneration	Melt-quenching	Glass	[313]
	46.14 SiO_2_–24.40 Na_2_O–26.91 CaO–2.55 P_2_O_5_ with 0, 0.41, 0.82, 1.23, and 1.65 Nb_2_O_5_ (mol%)	Bone regeneration	Melt-quenching	Glass	[314]
	46.14 SiO_2_–24.40 Na_2_O–26.91 CaO–2.55 P_2_O_5_ with 0, 0.41, 0.82, 1.23, and 1.65 Nb_2_O_5_ (mol%)	Bone regeneration	Melt-quenching	Glass	[315]
Niobium (Nb)	46.1 SiO_2_–24.5 Na_2_O–26.9 CaO–2.6 P_2_O_5_ with 0,1.0, 2.5, and 5.0 Nb_2_O_5_ (mol%)	Tissue engineering	Melt-quenching	Glass powder	[230]
	20 SiO_2_–24.5 Na_2_O–24.5 CaO–31B_2_O_3_ with 0, 2.5, 5, and 10 Nb_2_O_5_ (mol%)	Bone regeneration	Melt-quenching	Glass powder	[229]
	46.1 SiO_2_–24.5 Na_2_O–26.9 CaO–2.6 Nb_2_O_5_ (mol%)	Bone regeneration	Melt-quenching	Glass powder with a particle size range of 40–63 µm	[233]
Nitrogen (N)	55 SiO_2_–31.5 Na_2_O–13.5 CaO with 0, 1, 2, 3, and 4 Si_3_N_4_ (mol%)	Bone regeneration	Melt-quenching	Glass	[355]
	55 SiO_2_–31.5 Na_2_O–8.5 CaO–5 CaF_2_ with 0, 1, 2, 3, and 4 Si_3_N_4_ (mol%)	Bone regeneration	Melt-quenching	Glass	[356]
	55 SiO_2_–29 Na_2_O–13.5 CaO–2.5 P_2_O_5_ with 1, 2, 3, and 4 Si_3_N_4_ (mol%)	Bone regeneration	Melt-quenching	Glass	[357]
	45 SiO_2_–24.5 Na_2_O–24.5 CaO–6 P_2_O_5_ with 0, 5.51, and 10.69 Si_3_N_4_ (wt.%)	Bone regeneration	Melt-quenching	Glass	[358]
Palladium (Pd)	80 SiO_2_–15 CaO–5 P_2_O_5_ (mol%) with addition of 0.46, 0.96, 1.20, and 2.30 % PdCl_2_	Catalytic oxidation of benzyl alcohol	Sol–gel	Mesoporous powder	[319]
Rubidium (Rb)	80 SiO_2_–15 CaO–5 P_2_O_5_ with *x* = 0, 1, 2, and 5 Rb_2_O (mol%)	Bone regeneration	Sol–gel	Mesoporous bioactive glass scaffolds with macropores in the size range 350–550 µm using the foam replica method	[142]
	90 SiO_2_–10 CaO with 0, 0.5, 1.5, and 2.5 Rb_2_O (mol%)	Bone regeneration	Sol–gel	Nanoparticles with a particle size range of 100–114 nm	[140]
	80 SiO_2_–15 CaO–5 P_2_O_5_ with 0, 0.5, 1, 3, 5 and 10 Rb_2_O (mol%)	Wound healing	Sol–gel	Nanoparticles with a particle size range of 350–430 nm	[141]
Selenium (Se)	60 SiO_2_–36 CaO–4 P_2_O_5_ with 0, 1, 3, and 5 SeO_3_ (mol%)	Bone regeneration	Sol–gel	Mesoporous powder with a particle size around 400 nm	[376]
	80 SiO_2_–15 CaO–5 P_2_O_5_ with 0 and 5 SeO_3_ (mol%)	Bone tumor therapy	Sol–gel	Mesoporous powder with a surface area range of 200–350 m^2^/g and a mesopore size range of 3–5 nm	[374]
	40 SiO_2_–43 CaO–12 P_2_O_5_–5 MgO with 0, 2, 4, 6, and 8 SrO, and 0, 2, 3, and 4 SeO_3_ (mol%)	Bone regeneration	Sol–gel	Mesoporous powder with a particle size range of 265–318 nm	[390]
	45 SiO_2_–24.5 Na_2_O–24.5 CaO–6 P_2_O_5_ with 0.75, 1.5, 3, and 6 SeO_2_ (wt.%)	Bone cancer therapy	Melt-quenching	Glass powder	[375]
Tantalum (Ta)	80 SiO_2_–15 CaO–5 P_2_O_5_ with 0, 0.5, 5, and 10 Ta_2_O_5_ (mol%)	Tissue engineering	Sol–gel	Mesoporous powder with a particle size less than 45 µm	[162]
	58 SiO_2_–37 CaO–5P_2_O_5_ with 0, 0.2, 0.4, 0.6, 0.8, and 1 Ta_2_O_5_ (mol%)	Bone regeneration	Sol–gel	Glass powder	[173]
	20 SiO_2_–24.5 Na_2_O–24.5 CaO–31 B_2_O_3_ with 0.5, 1, 2, and 3 Ta_2_O_5_ (mol%)	Bone regeneration	Melt-quenching	Glass powder	[174]
Tellurium (Te)	26 Na_2_O–21 CaO–3 P_2_O_5_–50 TeO_2_ (mol%)	Bioactive implants	Melt-quenching	Glass powder with a particle size range of 75–150 µm	[361]
	48.6 SiO_2_–16.7 Na_2_O–34.2 CaO–0.5 P_2_O_5_ with 0, 1, and 5 TeO_2_ (mol%)	Bone regeneration	Melt-quenching	Glass powder with a particle size of less than 25 µm	[366]
Tin (Sn)	(35–40) P_2_O_5_–(40–60) SnCl_2_ with 5, 10, 15, and 20 SnCl_2_ (mol%)	Nuclear medicine	Melt-quenching	Glass	[350]
Tungsten (W)	44.7 SiO_2_–24.9 Na_2_O–24.9 CaO–5.5 P_2_O_5_ with 0, 1, 2, 3, and 4 WO_3_ (wt.%)	Radiation shielding materials	Melt-quenching	Glass	[320]
	5.50 Na_2_O–18.50 CaO–11.10 K_2_O–4.60 MgO–3.70 P_2_O_5_–56.60 B_2_O_3_ with 0, 0.5, 1, 2, and 4 WS_2_ (wt.%)	Radiation shielding materials	Melt-quenching	WS_2_ nanoparticle-containing bioactive glass composites	[321]
	75 B_2_O_3_–25 Li_2_O with 0, 1, 3, 5, and 7.5 WO_3_ (mol%)	Radiation shielding materials	Melt-quenching	Glass	[322]
Vanadium (V)	5.50 Na_2_O–18.50 CaO–11.10 K_2_O–4.60 MgO–3.70 P_2_O_5_–56.60 B_2_O_3_ with 0.5, 1, and 3 V_2_O_5_ (wt.%)	Bioimaging	Melt-quenching	Glass powder with a particle size of around 3.66 µm for 3 wt.% V_2_O_5_	[269]
	5.50 Na_2_O–18.50 CaO–11.10 K_2_O–4.60 MgO–3.70 P_2_O_5_–56.60 B_2_O_3_ with 0.5, 1, and 3 V_2_O_5_ (wt.%)	Medical radiation	Melt-quenching	Glass powder	[270]
	5.50 Na_2_O–18.50 CaO–11.10 K_2_O–4.60 MgO–3.70 P_2_O_5_–56.60 B_2_O_3_ with 0.5, 1, and 3 V_2_O_5_(wt.%)	Soft tissue repair and in wound healing	Melt-quenching	Glass powder with a particle size of around 14 µm and scaffolds with an average pore size of 500 µm using foam replication method	[268]
	5.50 Na_2_O–18.50 CaO–11.10 K_2_O–4.60 MgO–3.70 P_2_O_5_–56.60 B_2_O_3_ with 0.5, 1, and 3 V_2_O_5_ (wt.%)	Bone regeneration	Melt-quenching	Glass powder with a particle size of around 2 µm and scaffolds with an average pore size of 100–500 µm using foam replication method	[266]
	57.2 Si–35.3 Ca–7.5 P with 0, 0.71, 2.78, and 6.67 V (mol%)	Bone regeneration	Sol–gel	Mesoporous powder with a specific surface area range of 647–349 m^2^/g	[271]
Zirconium (Zr)	53 SiO_2_–6 Na_2_O–20 CaO–4 P_2_O_5_–12 K_2_O–5 MgO with 0, 0.5, 1.0, 1.5, and 2.0 ZrO_2_ (wt.%)	Bone regeneration	Melt-quenching	Glass powder	[197]
	22 Na_2_O–24 CaO–46 P_2_O_5_–8 ZnO with 0, 0.1, 0.3, 0.5, and 0.7 ZrO_2_ (mol%)	Bone regeneration	Melt-quenching	Glass parts with dimensions 1.5 cm × 1.5 cm × 0.2 cm	[175]
	60 SiO_2_–36 CaO–4 P_2_O_5_ with 0, 5 and 10 ZrO_2_ (mol%)	Bone regeneration	Sol–gel	Glass powder	[198]
	60 SiO_2_–31 CaO–4 P_2_O_5_–5 ZrO_2_ with 0, 2, 4, and 6 ZnO (mol%)	Bone regeneration	Sol–gel	Glass powder	[391]

## Rare earth elements-containing bioactive glasses

The incorporation of biologically active ions, including less-common ions, provides BG matrices with additional biological functionalities, therapeutic effects, and physical properties, for example, induction of hydroxyapatite formation, enhanced differentiation and proliferation of bone-forming cells, stimulating effects on angiogenic growth factors and improvement in mechanical properties [41]. Several studies have reported the use of rare earth elements in BGs to achieve different biological and functional properties. In this section, the effects of the incorporation of rare earth elements in different types of BGs are discussed.

### Europium (Eu)

Eu is a rare earth element that is not naturally present in the human body; however, as other elements, it can be incorporated into the body via ingestion of food and inhalation of dust particles. Normally these elements are naturally eliminated, but small amounts may deposit in organs. Traces of Eu have been found in brain tissue and kidney stones [55]. Due to the luminescent properties of Eu^3+^ ions, silicate and phosphate bioactive glasses doped with europium (Eu-BGs) have been designed for applications in drug delivery systems [56–59], cell imaging [60–67], optical devices [68], and bone and skin regeneration [69–74]. Eu-BGs were shown to emit strong red luminescence features at 590 nm and 612–616 nm when exposed to UV radiation [56, 69, 70]. In other studies, the intensity of emission was found to increase as the fraction of europium ions increased [69, 70]. The change in luminescence intensity of Eu^3+^ has been monitored to track the release of ibuprofen (IBU) [56, 58]. Fan et al. [58] observed the IBU release process using luminescence functionalized Eu-doped mesoporous bioactive glasses (Eu-MBGs) in the system SiO_2_–CaO–P_2_O_5._ The release of IBU from Eu-MBG in SBF increased the photoluminescence intensity of Eu^3+^ at 590 and 621 nm, reaching the highest value when IBU was completely removed. The quenching effect was weakened by the release of IBU, resulting in the increase of emission intensity [56, 58]. Moreover, Huang et al. [59] showed that the IBU release rate of Eu-doped mesoporous bioactive glass nanofibers (MBGNFs) with 5 mol% Eu^3+^ (or Tb^3+^) in the system 70 SiO_2_–25 CaO–5 P_2_O_5_ (mol%) was more rapid than for IBU-loaded MBG due to the disordered nanoporous channels present in the nanofibers. Zhang et al. [57] observed that increasing concentration of Eu in MBG nanospheres with composition 60 SiO_2_–(36–*x*) CaO–*x* Eu_2_O_3_–4 P_2_O_5_, *x* = 0.5, 1, and 2 mol%, changed the size, morphology, and pore structure of mesoporous silica supporting a controlled release of doxorubicin (DOX), a drug used for cancer treatment [57]. Xue et al. [60] demonstrated that fluorescent Eu ions in BG nanoparticles (80 SiO_2_–16 CaO–4 P_2_O_5_ mol%) were used to mark living murine calvaria-derived pre-osteoblastic (MC3T3-E1) cells for in vitro cytotoxicity studies with high red fluorescence and low background noise. Besides, Wu et al. [70] investigated the degradation of Eu-MBGs scaffolds (80 SiO_2_–15 CaO–5 P_2_O_5_, mol%) using a spectrofluorimeter to measure luminescence intensity at 615 nm. Also, they detected in vivo new bone formation in a bone defect promoted by Eu ions release (wavelength of 610 nm), indicating that Eu addition can have also a biological effect, as discussed next.

Eu-BGs have shown bioactive behavior in SBF [57, 60]. Eu incorporation in BG nanoparticles had no significant effect on apatite mineralization [60], although the morphology of the formed apatite layer changed as the doping Eu content raised [57]. Moreover, Wu et al. observed that ionic dissolution products of Eu-containing MBGs (5 mol%) at varying concentrations (from 6.25 to 100 mg/ml) facilitated proliferation and osteogenic differentiation of bone marrow stromal cells (BMSCs) by upregulating the expression of osteogenic genes (Runx2, COL1, OPN, OSX, and BSP) and by inducing ALP activity (6.25 and 25 mg/ml). However, the ALP activity decreased when the glass concentration was increased to 100 mg/ml. These results were compared to a control group that did not have conditioned medium. Similarly, europium-doped mesoporous silica nanospheres (Eu-MSNs) have been shown to substantially upregulate osteogenic markers (ALP, OPN, OCN, COL1, and Runx2) of BMSCs and to enhance the expression levels of CD31, PDGFRα/β, VEGFR1/2, and MMP9 angiogenic makers of human umbilical vein endothelial cells (HUVECs) indicating the promotion of both osteogenic and angiogenic differentiation [69]. The addition of europium also had positive therapeutic effects on pro-inflammatory macrophage cells (RAW 264.7) treated with Eu-MSN (0.2 mg/ml), resulting in reduced pro-inflammatory genes IL-18, IL-6, IL-1 β, OSM MyD88, Ticam1, and Ticom2 [69]. In addition, 2 mol% Eu-doped MSN and Eu-free MSN suspensions at a concentration of 0.2 mg/ml showed no cytotoxic effect on RAW 264.7 cells, while Eu-doped MSN induced macrophage proliferation. In contrast, non-doped MSN had no effect on macrophage proliferation [69]. Similarly, other studies have shown that Eu-BG had no cytotoxic effect on MC3T3-E1 cells at concentrations ranging from 40 to 250 µg/ml [60] and osteosarcoma MG 63 cells at different concentrations (between 50 and 200 µg/ml) compared to undoped BG [57]. Other studies have reported the possible in vitro cytotoxicity of Eu-containing BGs [57, 60, 69]. Moreover, in vivo studies of Eu-doped MSN have demonstrated that Eu accelerated the formation of new bone in a rat defect site after between 4 and 12 weeks of implantation [69, 70] and it promoted new blood vessels growth, collagen deposition, and re-epithelialization at the wound site [69].

### Holmium (Ho)

It has been reported that holmium may have an influence on accelerating metabolism in humans [75]. In addition, Poniedzialek et al. [76] investigated the possible presence of Ho in human colostrum milk, developed at the first stage of breast milk. In the field of BGs, Ho has been used mainly in silicate-based systems [77–79]. For example, sol–gel-derived holmium-doped 58S bioactive glasses (Ho-BGs) with compositions 58 SiO_2_–33 CaO–9 P_2_O_5_–*x* Ho_2_O_3_ (*x* = 1.25, 2.5, and 5 wt.%) have been shown to promote the proliferation of MC3T3-E1 cells in relation to the concentrations of Ho_2_O_3_ [78]. Moreover, the addition of Ho was shown to significantly affect the dissolution behavior due to the presence of Si-O-Ho covalent bonds in the glass network, which reduced the dissolution rate of the glass without slowing down the bioactive behavior. Ho-BG powders exhibited apatite-like structures on the surface for all Ho_2_O_3_ concentrations [78]. These results showed that Ho-containing BGs could be an interesting alternative for bone tissue regeneration. Zambanini et al. [79] investigated 58S BGs (58 SiO_2_–33 CaO–9 P_2_O_5_) containing various amounts of Ho_2_O_3_ (1.25, 2.5, 3.75, and 5 wt.%) incorporated into a Poloxamer 407 hydrogel (20 wt.%) for brachytherapy applications [80]. The hydrogel was integrated with Ho_2_O_3_ containing BG, and it was found that the glass particles greatly influenced the hydrogel self-assembly potential. In contrast, the hydrogel viscosity was significantly reduced at 37 °C. Furthermore, the hydrogel containing 5 wt.% Ho-BG particles enhanced the proliferation of MC3T3-E1 cells [79]. Clearly, given the scarcity of investigations, the potential of Ho-BGs in tissue engineering applications remains unexplored.

### Gadolinium (Gd), ytterbium (Yb), and thulium (Tm)

**Fig. 3:**
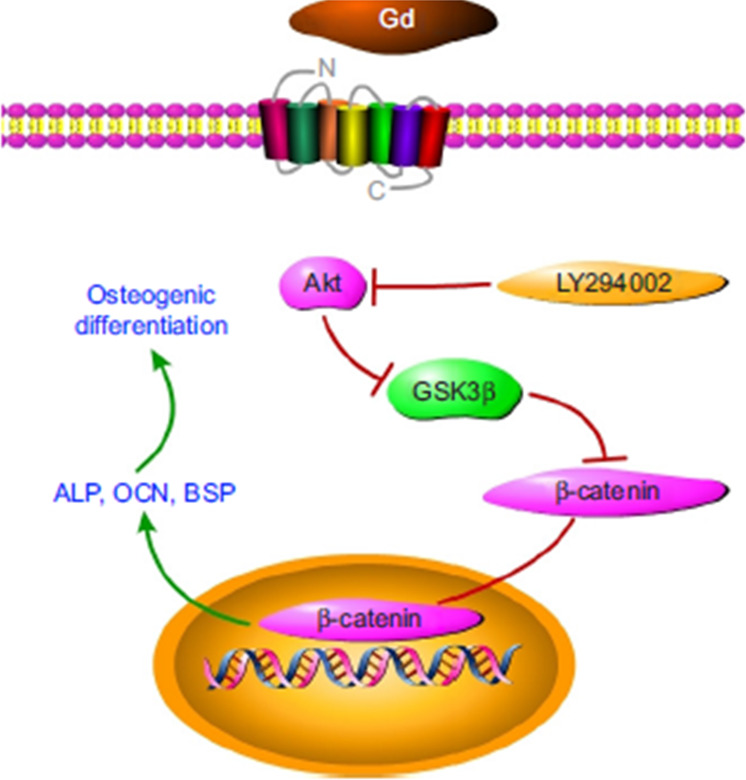
Schematic diagram showing Gd dopant activation of the Akt/GSK3β signaling pathway [89]. Reproduced according to Creative Commons license (CC BY-NC 3.0)

Gd has been widely used in contrast agents for magnetic resonance imaging aimed to be eliminated naturally from the body; however, it has been shown that Gd could deposit in the brain and bones [55, 81, 82]. Similarly, Yb belongs to the lanthanide series of elements that are not naturally present in the human body. This element is highly used in optics and as a doping agent to increase the mechanical properties of stainless steel. Furthermore, Yb has been reported to accumulate in soils and water mainly due to petrol producing industries or discarded household equipment [83]. Silicate-based bioactive glasses doped with gadolinium (Gd-BG) and ytterbium (Yb-BG) have been investigated [84–88] due to the characteristic features that these elements offer for biomedical applications in the fields of brachytherapy, luminescence-based imaging, and magnetic resonance imaging [84]. In vitro bioactivity and biological studies have been performed on Gd and Yb containing BGs (of composition 47.28 SiO_2_–31.39 Na_2_O–15.33 CaO–6 P_2_O_5_ with 2.5 Gd_2_O_3_ or Yb_2_O_3_ wt.%), resulting in calcium phosphate deposition after 1 day of immersion in SBF and a lower dissolution behavior compared to the reference glass owing to the covalent character of the Si-O-Gd and Si-O-Yb bonds. In terms of cytocompatibility, the authors reported viability higher than 80% of mesenchymal stem cells derived from deciduous teeth (SHEDs) [85]. Moreover, gadolinium has been shown to have favorable therapeutic effect on osteoinductivity. For example, Zhu et al. [89] demonstrated that Gd-BG mesoporous microspheres in chitosan scaffolds facilitated the proliferation, differentiation, and expression of ALP activity, OCN, and BSP via Akt/GSK3β activation of human bone marrow-derived mesenchymal stem cells (hBMSCs). The AKT/GSK3 signaling pathway is crucial for the survival of human pluripotent stem cells (Fig. 3). Similarly, by triggering the Wnt/-catenin signaling pathway, Gd-doped mesoporous calcium silicate containing scaffolds facilitated the osteogenic potential of rBMSCs [90]. With Gd incorporation in BG, the expression of osteogenic markers such as ALP activity, Runx2, and COL-1 increased [89, 90]. Furthermore, in vivo studies in a mouse model demonstrated that Gd-BG incorporation in chitosan scaffolds promoted rapid and significant newly formed bone and collagen deposition in a calvarial defect after 8–12 weeks implantation [89, 90].

Thulium has also been used with ytterbium to produce co-doped sol–gel-derived silica glass nanoparticles with different ratios of Tm_2_O_3_ and Yb_2_O_3_ for biological testing, bioimaging, and drug delivery systems [91]. Nanoparticles with basic SiO_2_-CaO, containing Tm_2_O_3_ (0.15, 0.3, or 0.5 mol%) and Yb_2_O_3_ (0, 1, 2, 3, or 4 mol%), showed amorphous structure for lower dopant concentrations, while crystallization of calcium silicate was detected for the higher amounts of Tm_2_O_3_ and Yb_2_O_3_. The authors concluded that samples with 0.3% Tm_2_O_3_ and 4% Yb_2_O_3_ are promising due to their higher emission intensity and single exponential decay time compared to the other tested concentrations.

### Samarium (Sm)

Sm, an element that has in principle no natural biological role, has been widely used as a radiopharmaceutical to treat cancer in bones [92]. Sm-doped bioactive glasses (Sm-BG) have shown photoluminescence properties characteristic of Sm^3+^ ions and have been described as potential material for cancer treatment [93–95]. Baranowska et al. [96] used the luminescent properties (at 601 and 648 nm) of bioactive 45S5 BG fibers doped with Sm^3+^ to investigate the degradation behavior of the fibers. Furthermore, in vitro formation of apatite-like structures on Sm-BG substrates was observed after incubation in SBF by Ershad et al. [97]. The authors found that adding Sm_2_O_3_ to BGs up to a concentration of 3 wt.% increased the formation of hydroxycarbonate-apatite (HCA) layer on the surface after 21 days. Furthermore, Sm-BGs exhibited enhanced mechanical properties. Young’s modulus (76.36–78.89 GPa), shear modulus (30.25–31.95 GPa), and bulk modulus of Sm-containing 45S5 BGs increased with increasing concentration of Sm_2_O_3_ [97]. Poisson’s ratio, on the other hand, decreased as the concentration of Sm_2_O_3_ increased. [97]. In addition, Zhang et al. [98] investigated the potential use of samarium (0.5–1 mol%) doped mesoporous BG and alginate-containing microspheres for drug delivery applications. The drug (DOX) was loaded in the microspheres with varying amounts of Sm. The release of DOX was proportional to the Sm doping concentration due to the higher dissolution rates proportional to the Sm concentration [98].

Morais et al. [99] investigated melt-derived samarium-doped phosphate glasses (15 CaO–10 Na_2_O–15 CaF_2_–65 P_2_O_5_, with Sm_2_O_3_ ranging from 0.5 to 2 mol%) and hydroxyapatite to produce composites (BG-HA). A proportion of 2.5 wt.% Sm-doped BG to 97.5 wt.% hydroxyapatite was used to make the composites. XRD analysis showed crystalline phases characteristic of hydroxyapatite and samarium oxide. Moreover, the addition of Sm^3+^ ions in the composite increased surface hydrophilicity and flexural strength compared to Sm-free BG-HA. The highest concentration of Sm in the BG-HA composites affected in vitro the antibacterial activity and cytocompatibility behavior. Consequently, BG-HA doped with 2 mol% Sm_2_O_3_ showed the best antibacterial performance against *Staphylococcus aureus* and *S. epidermidis* besides higher proliferation of MG 63 cells and upregulation of relevant osteogenic markers (Runx2, ALP, BMP-2, and OC) [99].

### Yttrium (Y)

Yttrium has been used in the clinic in cancer treatment [92]. Various studies have investigated the incorporation of yttrium in BGs for applications in different fields including radiotherapy, dentistry, and bone tissue engineering [100–109]. Yttrium-doped glasses (Y-BGs) have reported good chemical durability and stability in in vivo radiotherapy settings [110]. Erbe and Day [111] investigated the effect of the processability of Y-containing glasses (17 Y_2_O_3_–19 Al_2_O_3_–64 SiO_2_ mol%) on their chemical durability. Sol–gel-derived and melt-derived Y-doped glass microspheres have shown higher chemical durability than bulk particles due to their large surface area. A SiO_2_-rich surface on the microspheres triggered surface corrosion after 4 weeks in DI water or 12 M HCl. Moreover, the glass durability after the addition of 4.68 mol% of Y_2_O_3_ in the BG composition (62.35 SiO_2_–1.0 P_2_O_5_–15.85 Na_2_O–20.8 CaO mol%) was investigated by Christie et al. [112]. Molecular dynamics simulations revealed that the substitution of 4.68 mol% Y_2_O_3_ for CaO in the BG composition led to an increased dissolution rate compared to Y-free BG due to the generation of a fragmented silicate network, causing a lower network connectivity and glass durability. The yttrium release rate was computed using site-selectivity and clustering of yttrium cations [112]. Arafat et al. [113] investigated the degradation rate after the incorporation of Y_2_O_3_ (3 and 5 mol%) in phosphate-based glasses (substitution for Y_2_O_3_/Na_2_O) in phosphate buffer saline and ultra-pure water (Milli-Q water) at 37 °C over 28 days. The results showed a reduced degradation rate with increasing Y_2_O_3_ content in the glass system 45 P_2_O_5_–25 CaO–30 Na_2_O (mol%). In addition, Y-doped BGs have also exhibited bioactive behavior. Tesfay et al. [105], for example, observed that Y-containing 58S BG led to rapid apatite-like formation after 6 h in SBF. Recent work has also shown that replacing B_2_O_3_ with 1 wt.% Y_2_O_3_ in the glass composition 53 B_2_O_3_–20 CaO–12 K_2_O–6 Na_2_O–5 MgO–4 P_2_O_5_ (wt.%) had a greater effect on the proliferation and migration of adipose stem cells (ASCs) in an α-minimal essential medium in vitro [114].

### Lanthanum (La)

La is a rare earth element that is present at low levels in drinking water and food. It has been reported to have chemical similarities to Ba, Sr, and Ca and has been recently investigated to replace calcium-based phosphate binders needed in patients with kidney failure to reduce cardiovascular calcification [115]. Therefore, tracing the accumulation of La in the body has become an important aspect for such applications, being bone the main accumulation site reported so far [81], next to breast milk [76] and brain tissue [55]. Lanthanum has been used to modify the properties of silicate and phosphate BGs [74, 116–122]. Lanthanum-doped bioactive glasses (La-BGs) containing chitosan composite scaffolds significantly improved osteoblast performance in terms of promoting the proliferation and osteogenic differentiation of BMSCs by upregulating expression levels of osteogenic markers (ALP, OCN, BMP-2, and Runx2) and raising the protein expression of RK in comparison to the scaffold without La doping [117]. In contact with HUVECs, La-BG-based scaffolds significantly induced the expression levels of b-FGF, vascular endothelial growth factor (VEGF), PDGF, and qRT-PCR compared to La-free BG scaffolds [117]. In vivo, the implantation of La-BG containing chitosan scaffolds in rat calvarial defects induced bone regeneration and new blood vessel formation after 8 weeks of implantation [117]. The addition of La_2_O_3_ (5 and 10 mol%) to phosphate glass nanoparticles provided a sustained delivery of the antibiotic ciprofloxacin for up to 28 days; on the other side, pure glass nanoparticles showed sustained drug release for 20 days [118]. The viability of fibroblast baby hamster kidney cells (BHK) after exposure to La containing nanoparticles exhibited a lanthanum oxide concentration dependency. The cell viability increased from 80 to 93% with increasing La concentration (from 0 to 10 mol%) [118]. Incorporation of lanthanum ions in combination with copper ions in BG facilitated the formation of a hydroxyapatite layer on the BG surface after soaking in SBF [123], suppressed C13895 lymphoblast cytotoxicity [123], and improved mechanical properties [124]. In addition, Jodati et al. [125] found multiple advantages of magnesium-lanthanum dual doped BGs (1 wt.% La) in bone regeneration applications, with the glasses exhibiting increased bioactivity in terms of apatite formation ability and biocompatibility with SAOS-2 cells (human osteosarcoma).

### Terbium (Tb) and erbium (Er)

Tb and Er have been used in medical imaging applications [75]. Bioactive glasses doped with terbium (Tb-BG) have been recently studied for biomedical applications because of their attractive properties, such as bioactivity, biocompatibility, biodegradation, and non-toxicity [126–129]. Wang et al. [130] investigated the influence of Tb on the apatite formation ability of mesoporous BG nanospheres (base composition: 79.5 SiO_2_–15 CaO–5 P_2_O_5_ mol%). It was reported that the incorporation of Tb_2_O_3_ (0.5 and 1 mol%) led to enhanced hydroxyapatite formation after immersion in SBF for 3 days. The hydroxyapatite nucleation on the surface of Tb-MBG nanospheres was seen to increase by the release of Ca^2+^ and Tb^3+^ ions. Furthermore, by varying Tb concentrations, it was possible to tailor DOX release [130]. Moreover, Tb-MBG nanospheres showed a nontoxic effect on MC3T3-E1 cells in indirect cell culture experiments at concentrations of 50 and 100 µg/ml [130]. Huang et al. [59] also evaluated the biocompatibility of Tb^3+^ (and Eu^3+^) containing MBGNFs using the MTT assay at different MBGNF concentrations (3.125, 6.25, 12.5, 25, 50, 100, and 200 µm/ml). In all conditions, the viability of L929 fibroblast cells was higher than 90%, suggesting no cytotoxic effect of Tb^3+^ (or Eu^3+^) doped MBGNF. Under ultraviolet irradiation, Tb-MBGNF and Eu-MBGNF showed luminescence properties at 544 and 614 nm, respectively [59].

Furthermore, Li et al. [128, 131] investigated co-doped BGs with Er and Yb to provide conventional BGs with luminescence properties for biological labeling and drug delivery applications. Er_2_O_3_ (0.79–3.52 wt.%) and Yb_2_O_3_ (6.36–28.12 wt.%) were incorporated in Ca-Mg-Si BGs [131], as well as Er_2_O_3_ (1–2 wt.%) and Yb_2_O_3_ (9–18 wt.%) in CaSiO_3_ [128]. In both investigations, bioactivity studies showed that co-doped BGs exhibited apatite precipitation in interaction with SBF after 14 days [128, 131]. Furthermore, these materials did not show cytotoxic behavior to MC3T3-E1 cells, human dermal fibroblasts cells (HDFs), and HUVECs [128, 131]. In addition, culture of HDFs and HUVECs with the ionic extracts of the Er^3+^ and Yb^3+^ co-doped Ca-Mg-Si BGs showed enhanced cell proliferation, expression of angiogenic genes and cell migration in comparison to non-doped glasses [131].

In a recent study, Deliormanli et al. [132] synthesized sol–gel-derived 13–93 BG doped with Er_2_O_3_ (1–5 wt.%) and Tb_2_O_3_ (1–5 wt.%) as well as co-doped BGs (Er_2_O_3_ and Tb_2_O_3_ from 0.5 to 2.5 wt.%). These BGs were successfully shaped into fibers via electrospinning. The addition of Er^3+^ and/or Tb^3+^ to the BG structure has been shown to affect the photoluminescence and decay times of the BG particles and nanofibers significantly. Consequently, the authors reported an effect of the BG morphology on the luminescence emission intensity and decay kinetics. The BG particles exhibited stronger emission intensity while the electrospun nanofibers longer decay times. Furthermore, the incorporation of Er^3+^ and/or Tb^3+^ into 13–93 BGs did not have an effect on hydroxyapatite formation after incubation in SBF for 30 days. The results were comparable to non-doped 13–93 BG particles and nanofibers, even at the highest doping concentration.

### Dysprosium (Dy)

Dysprosium-containing glasses have been investigated as biodegradable radiation delivery vehicles for the treatment of rheumatoid arthritis [133]. Microspheres made of lithium borate glasses-containing dysprosium oxide have been reported in studies of Day et al. [133, 134]. Melt-derived microspheres of composition 30 Dy_2_O_3_, 8.8 Li_2_O, and 61.2 B_2_O_3_ (in wt.%) have been further processed by a nonuniform reaction process with phosphate solutions to obtain highly porous dysprosium phosphate microspheres suitable for controlled delivery of drugs and radiation therapy. Moreover, Pătcaş et al. [135] investigated the structural changes of sol–gel silicate glasses containing dysprosium and iron after different thermal treatments (composition: 50 SiO_2_, 30 CaO, 10 Fe_2_O_3_,10 Dy_2_O_3_ in mol%). Glasses treated at 500, 800, and 1200 °C exhibited decreasing surface area values at increasing temperature. Furthermore, nanocrystalline magnetite, hematite, and wollastonite phases were detected in the samples treated at 800 and 1200 °C, which could lead to bioactive materials for applications on radiotherapy and hyperthermia.

## Bioactive glasses doped with other elements

Elements belonging to different classifications in the periodic table such as alkali metals, transition metals and non-metals have also been incorporated in BGs. Table 2 summarizes the glass compositions and applications of the described systems and specific examples are described in the following sections.

### Alkali and alkaline-earth metals

#### Rubidium (Rb)

Rubidium (Rb) is an important element present in human and animal tissues [136]. It is found in human organs such as the liver, kidneys, cerebrum, cerebellum, heart, pancreas, and spleen [137, 138]. The application of Rb-containing BGs has been focused on bone regeneration and wound healing [139–141]. For example, incorporation of 0.5, 1.5, and 2.5 mol% Rb_2_O in bioactive glass nanoparticles (Rb-BGNs) of composition 90 SiO_2_–10 CaO (mol%) with varying CaO:Rb_2_O ratio was shown to increase the apatite-forming ability in SBF compared to Rb-free BGNs [140]. The greater ionic radius of Rb (1.48) relative to Ca (0.99) and Si (0.42) contributed to an open silica network structure and accelerated the release of Rb^+^ and Ca^2+^ in SBF, leading to a higher apatite deposition rate [140]. The authors have discovered that varying Rb_2_O content had no significant effect on morphology, scale, shape, chemical composition, and structure of the sol–gel-derived BG [142]. Similarly, Rubidium-containing mesoporous bioactive glasses (Rb-MBGs) shaped into scaffolds (80 SiO_2_–(15-*x*) CaO–5 P_2_O_5_–*x* Rb_2_O with *x* = 0, 1, 2, and 5 in mol%) were shown to exhibit enhanced bioactivity and promoted osteogenesis and angiogenesis [142]. Biomimetic surface mineralization of Rb-MBG scaffolds was assessed in SBF immersion resulting in the formation of a nanostructured apatite phase on the surface upon contact with SBF for 3 days. In terms of proliferation and osteogenic differentiation of human mesenchymal stem cells, the ALP activity and expression of COL-1, VEGF HIF-1α, and Wnt/ß-catenin signaling, significantly increased with Rb addition compared to Rb-free MBG scaffolds [142]. Similarly, the antibiotic enoxacin (ENX) was loaded into Rb-MBG scaffolds to explore the ability of the constructs to act as drug delivery carriers and specifically to provide antibacterial effect [142]. It was found that 5 mol% Rb-doped MBG (5Rb-MBG) scaffolds and ENX-loaded 5Rb-MBG scaffolds reduced the viability of *Escherichia coli* and *S. aureus* compared to bare MBG scaffolds [142]. Rb-doped bioactive glass nanospheres (Rb-BGNs) for skin regeneration and wound healing applications have been examined as alternative biomaterials for soft tissue regeneration [58]. He et al. [141] reported that BGNs with Rb content greater than 3 mol% were toxic to HUVECs, fibroblasts, and HaCaTs cells, while BGNs with Rb contents less than or equal to 3 mol% were nontoxic to the same cells. Interestingly, the ionic dissolution products of Rb-BGNs stimulated vascular tubule formation in contact with HUVECs through angiogenesis-related gene expressions such as HIF-1α and VEGF, aided by growth-promoting molecules, for instance TGF-β1, FGF2, PDGF, and EGF, as well as by triggering the ERK and P38 signaling pathways [141]. In vivo studies revealed that Rb-BGNs loaded with EGF accelerated wound healing of rats and have potential as endothelial growth factor transport vehicles with high bioactivity [141].

#### Barium (Ba)

Barium is a trace element found in the human body (22 mg in a 70 kg adult) [143]. Most Ba is found in bones and smaller amounts are present in muscle, skin, connective tissue, and lungs. Similar to other elements, barium can enter the body through the air, food, and drinking water containing this element; however, the quantity of Ba in food and water is generally insufficient to cause health problems [143]. Dietary barium intake for adults has been reported in the range of 0.4–1.8 mg/day and exposure to 3–4 g of Ba has been found toxic [144]. Clinically, barium sulfate is used in screening treatments and x-ray images [144] and in the last years, it has been considered as a therapeutic ion since it has shown stimulative effects on bioactivity, antibacterial, and anti-inflammatory properties in BGs [9, 63, 87, 145–158]. Majumdar et al. [9] synthesized nanoparticles of Ba-doped bioactive glass with composition 44.85 SiO_2_–2.6 P_2_O_5_–24.3 Na_2_O–26.9 CaO–1.35 BaO (mol%) by sol–gel process. XRD analysis confirmed the amorphous nature of the bioactive glass containing BaO. Ba^2+^ doping showed a positive effect on the bioactive behavior exhibiting the formation of HCA after immersion in SBF for 1 day. It was reported that Ba^2+^ (radius = 135 pm) replaced Ca^2+^ (radius = 100 pm) in the glass network, causing the glass network to become less rigid, resulting in a higher dissolution rate and faster ion release, enhancing bioactivity through the formation of hydroxyapatite. The cytocompatibility of Ba-containing BG and 45S5 BG as control was assessed using glioblastoma (C6 cells) and granulocytic 466 origin (K562) cells. Both Ba-containing BG and 45S5 BG enhanced proliferation in both cell lines without causing cytotoxicity. Moreover, in the same study, the ability of Ba^2+^ to prevent the lipopolysaccharide-induced amplification of interleukin-6 (IL-6), tumor necrosis factor-α (TNF-α), and interleukin-10 (IL-10) was evaluated indicating the anti-inflammatory effect of this ion [9]. In another approach, Paliwal et al. [159] synthesized melt-derived Ba-doped 45S5 BGs (1.3 BaO mol%) and evaluated their effect on gastro-duodenal ulcers. After soaking in SBF on days 6 and 7, Ba-doped BGs exhibited higher pH values than 45S5 BG, indicating that Ba-containing BGs may have an enhanced antacid-like effect over 45S5 BG. In an in vivo study using a rat model, gastric ulcers were induced by various ulcerogens such as ethanol, aspirin, pyloric ligation, and acetic acid, besides duodenal ulcers were induced by cysteamine. BGs were suspended and administered at dose levels of 0.3, 1.0, and 3 mg/kg. The results of the study revealed that Ba-BGs enhanced cell proliferation in the pyloric-induced gastric model and produced a protective layer on gastric and duodenum epithelium in the ethanol-induced gastric ulcer model. Furthermore, it was concluded that Ba-45S5 BGs in the dose of 3 mg/kg prevented and healed gastric-duodenal ulcers induced by different ulcerogens [159]. For cancer hyperthermia applications, the combination of magnetic properties and bioactive behavior of Ba-containing BGs is gaining attention. Yazdanpanah et al. [154] investigated a CaO–P_2_O_5_–SiO_2_–BaO–Fe_2_O magnetic sol–gel-derived BG system. Apatite layer deposition on the glass surface was influenced by the addition of Ba and Fe to the glass composition (0–10 mol% of BaO and 0–15 mol% of Fe_2_O_3_). Bioactivity improved when BaO content increased; however, it declined as Fe concentrations increased. In addition, the Ba-containing BG was nontoxic to L929 mouse fibroblast cells. In another application, Zakaly et al. [160] investigated the nuclear radiation attenuation features of borosilicate glasses doped with barium as radiation shielding material. The melt-quenching technique was used to produce BGs with base composition: 50 B_2_O_3_–20 NaO–15 SiO_2_–10 CaO–5 Al_2_O_3_ (in wt.%) and increasing BaO content; from 0 to 30 wt.%. The density and hardness improved with increasing BaO content. XRD analysis confirmed that the incorporation of BaO did not affect the amorphous structure of the glasses. Furthermore, specific material features such as mass attenuation coefficient (MAC), linear attenuation coefficient (LAC), mean free path (λ), and half-value layer (X1/2) can be used to study the effective radiation shielding of materials. When 30 wt.% BaO was incorporated in the glass, the glass density increased (from 2.673 to 3.652 g/cm^3^) resulting in lower λ and X1/2 values, as well as higher MAC and LAC, indicating that there was a superior gamma shielding and enhanced transmission and optical bandgap. High-density glasses resulted in higher effective shielding than low-density glasses [160].

### Transition metals

#### Tantalum (Ta)

Ta has been known as a biocompatible metal with superior properties in terms of corrosion resistance and bioactivity, consequently it has been considered for surgical implants [161]. The addition of tantalum to bioactive glasses has been reported in different investigations [151, 162–172]. Silicate bioactive glasses produced by sol–gel in the system 58 SiO_2_–37 CaO–5 P_2_O_5_ (mol%) doped with 0.2–1 mol% tantalum pentoxide (Ta_2_O_5_) revealed a rapid in vitro acellular HCA deposition (6 h) after soaking in SBF. Doping with tantalum improved the ability of glasses to develop apatite-like structures at concentrations 0.2–0.6 mol%, but a retarding effect at higher Ta concentrations (0.8, and 1 Ta mol%.) was found. These glasses also showed an antibacterial effect against *S. aureus* and *E. coli*; these properties make Ta a promising therapeutic dopant in bioactive glasses for bone tissue engineering [173]. Nagrath et al. [162] reported the hemostatic properties of Ta-doped MBGs of composition 80 SiO_2_–15 CaO–5 P_2_O_5_ (mol%), in which various Ta_2_O_5_ concentrations were analyzed from 0 to 10 mol%. Ta supplementation showed hemostatic potential due to its negative zeta potential (–23 to –31 mv), which enhanced the intrinsic mechanism of blood plasma coagulation and promoted hemostasis by decreasing the active partial thromboplastin and prothrombin times. According to cytotoxicity evaluation, Ta-MBGs (Ta concentration of 0, 0.5, 1, and 5 mol%) did not have a negative effect on the viability of bovine fibroblast cells [162]. Moreover, the in vitro bioactivity and cytocompatibility of Ta-doped borosilicate BGs have also been reported [174], concluding that the addition of Ta from 0.5 to 3 mol% in borosilicate BGs had an influence on the bioactive behavior, resulting in lower bioactivity for higher concentrations of Ta (3 mol%), without affecting cell viability (MG 63 cells).

#### Zirconium (Zr)

Zirconium as zirconium oxide has been used in the biomedical field for dental [175] and bone implants due to its superior mechanical properties and cytocompatibility [176–195]. Enhancement in mechanical stability and hydroxyapatite formation in silicate, borate, and phosphate bioactive glasses has been observed by incorporating zirconium [183, 196–198]. Yadav et al. [197] reported that the addition of zirconium (up to 2.0 wt.%) in 13–93 bioactive glass resulted in a significantly faster dissolution rate and a higher pH of SBF solution dependent on the zirconium concentration. In order to facilitate bone tissue engineering, suitable mechanical properties of the scaffold materials are important. As reported by Kumar et al. [183], compressive strength values increased from 10 ± 2 to 19 + 2 MPa when ZrO_2_ nanoparticle content was increased from 0 to 0.2 g in 56 SiO_2_–34 CaO–10 P_2_O_5_ (mol%) bioactive glass scaffolds, leading to the formation of ZrSiO, ZrSiO_4_, Zr_2_O (PO_4_), and Ca(ZrO_3_) crystalline phases. These values are comparable to the compressive strength of human cancellous bone, which ranges from 1.5 to 45 MPa [199]. By raising ZrO_2_ concentration to 5 wt.%, the microhardness of melt-derived borosilicate bioactive glass (31 B_2_O_3_–20 SiO_2_–24.5 Na_2_O–24.5 CaO mol%) improved from 5.45 to 6.17 GPs, while the apatite-formation ability decreased [188]. ZrO_2_ has been shown to display strong antibacterial properties. According to Kumar et al. [183], Zr-BG scaffolds showed antibacterial activity against *S. aureus*, *E. coli*, and *Pseudomonas aeruginosa*, but only a weak effect on *Bacillus subtilis*. The biological behavior of Zr-containing 3D scaffolds with composition 60 SiO_2_–36 CaO–4 P_2_O_5_ mol% (58S BG) was investigated by Moghanian et al. [198]. After incubation for 7 and 14 days, 3D-porous 58S BG scaffolds containing 0–10 mol% ZrO_2_ stimulated MC3T3-E1 cell adhesion on the scaffold and enhanced cell proliferation at more prolonged periods of incubation. The ALP activity of MC3T3-E1 cells increased with the presence of ZrO_2_ in the 58BG scaffold at all time points. Interestingly, the glass containing 5 mol% Zr showed the highest ALP activity compared to the other BGs [198]. The non-cytotoxic effect of zirconium-doped bioactive glass (5–15 wt.% of nano ZrO_2_ powder) as thin film coatings on Cp-Ti substrates has also been investigated on MG 63 osteoblast cells [200]. Moreover, a recent study reported the advantages of 13–93 bioactive glass doped with zirconium (2 mol%) and silver oxide. Co-doping with Zr and Ag in 13–93 BG improved cytocompatibility of U2OS cells, antibacterial effects against *B. subtilis* and *E. coli*, and led to mechanical properties enhancement in terms of compression strength, elastic modulus, and flexural strength [201].

#### Niobium (Nb)

Therapeutic niobium ions have been shown to play an influencing role in bioactivity, biocompatibility, and mechanical properties of bioactive glasses and bioceramics for regenerating bone tissue [202–228]. Bioactive borosilicate glass (31 B_2_O_3_–20 SiO_2_–24.5 Na_2_O–24.5 CaO mol%) doped with niobium (Nb-borosilicate BG) has shown in vitro bioactivity in terms of hydroxyapatite forming ability when soaked in SBF solution after 7 days, exhibiting no cytotoxic effect on MG 63 cells. The ability to form an apatite layer and support cell viability was unaffected by different concentrations of Nb_2_O_5_ (0–10 mol%) [229]. Nevertheless, the bioactivity of Nb-doped BG needs further investigation. Lopes et al. [230] investigated 45S5 BG with 2.5 and 5 mol% concentrations of Nb_2_O_5_, which showed a delayed formation of HCA on the BG surface compared to both 45S5 BG and 1 mol% Nb_2_O_5_-doped 45S5 BG.

**Fig. 4:**
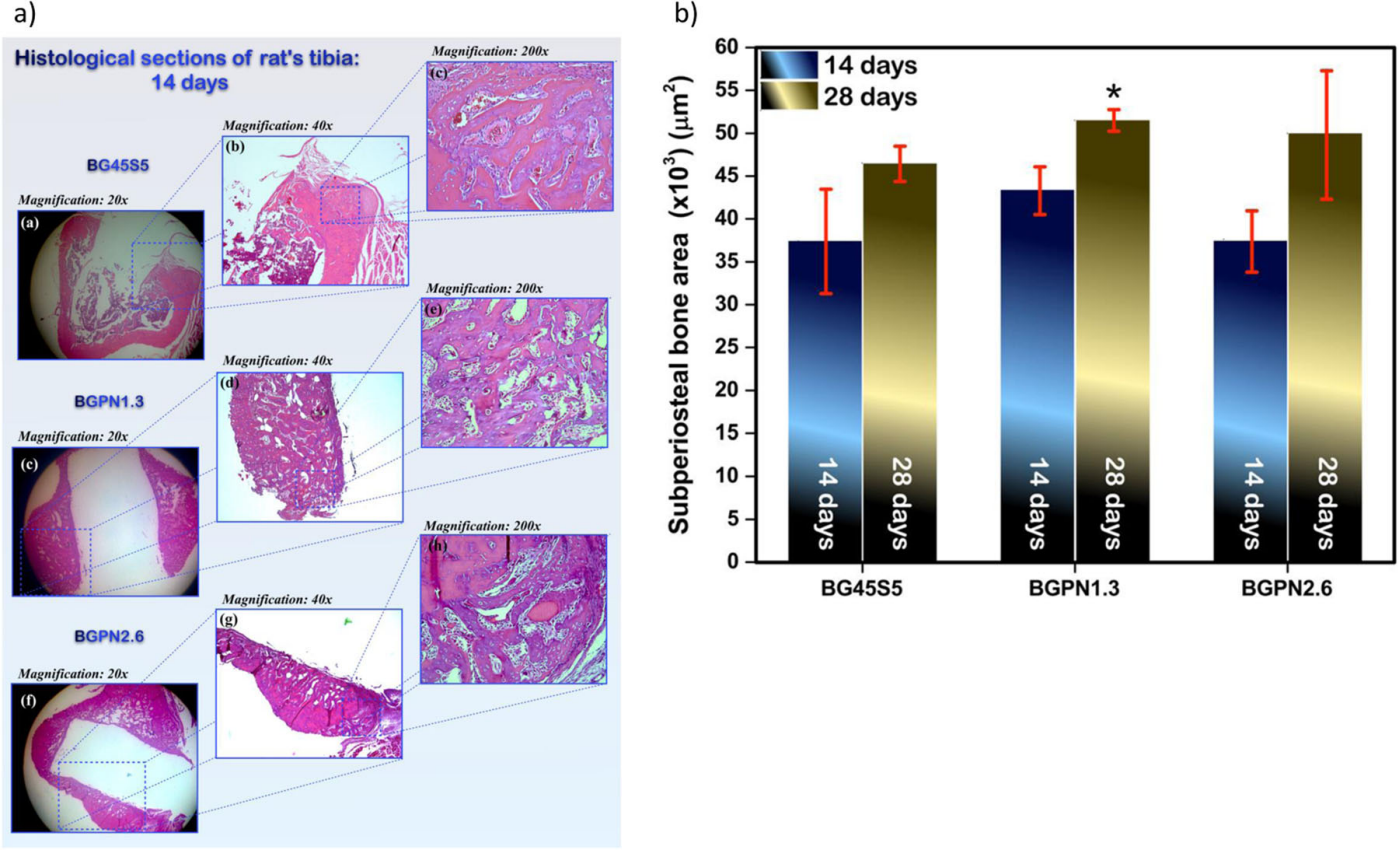
In vivo implantation of Nb-containing 45S5 BG rods: **a** subperiosteal new bone formation in rat tibia tissue defect after 28 days of implantation, hematoxylin & eosin staining, **b** growth area of subperiosteal bone in rats treated at different times [232]. Reproduced according to Creative Commons license (CC BY 4.0)

The presence of niobium in bioactive glasses could also promote osteogenic and angiogenic properties. In vitro cell studies have shown the cytocompatibility, osteostimulation, and osteoinduction of Nb-doped 45S5 BG [230]. In this study, Nb-substituted glasses had no negative effect on bone marrow-derived mesenchymal stem cells (BMSCs). Moreover, osteogenic differentiation of BMSCs was induced at concentrations of 1 and 2.5 mol% Nb_2_O_5_ in 45S5 BG after 21 days using a glass concentration of 10 mg/ml [230].

**Fig. 5:**
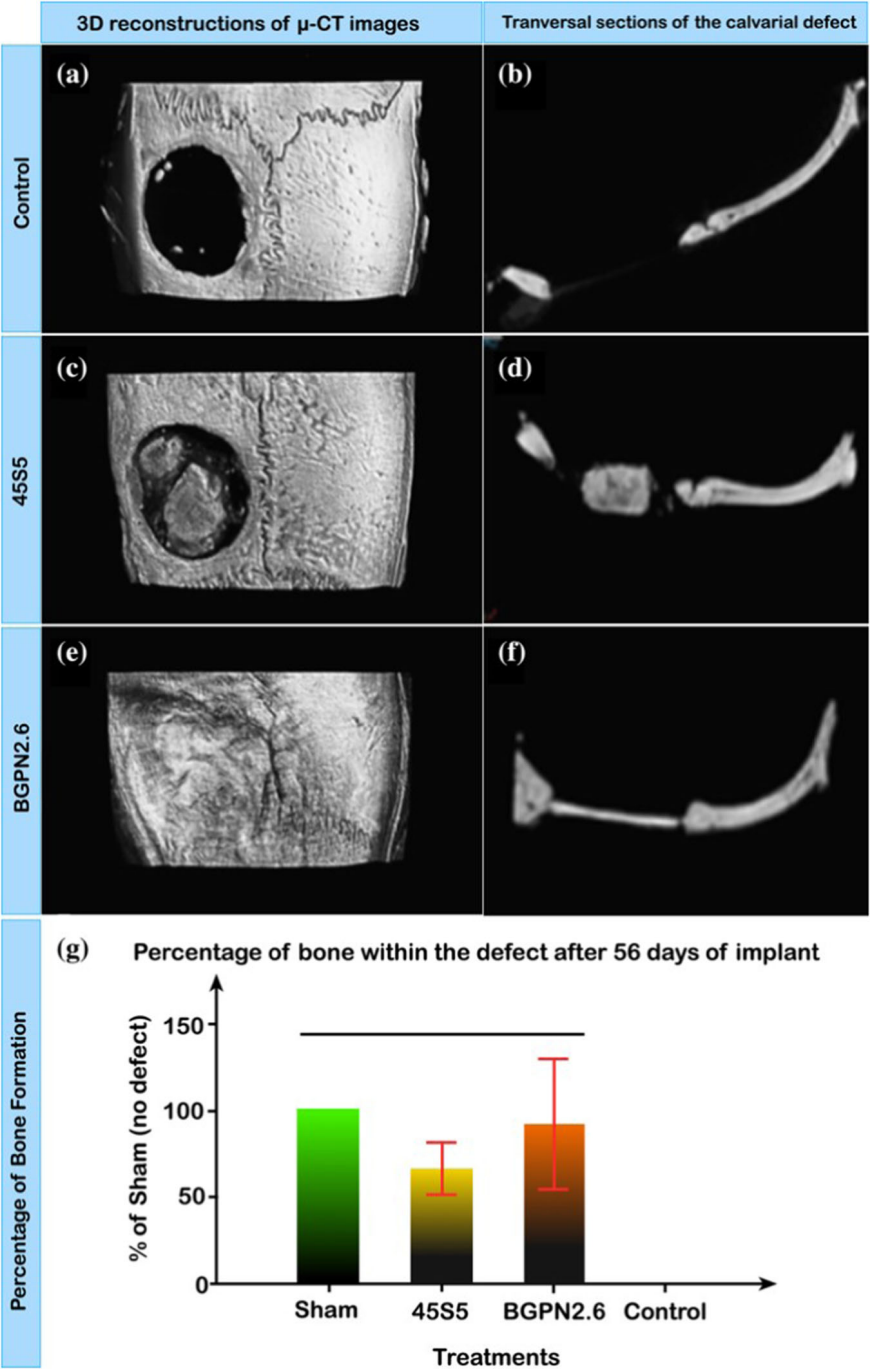
Microcomputed tomography images showing bone regeneration in a 5-mm critical-size defect in rat calvaria after 56 days [233]. Reproduced with permission from John Wiley and Sons

In similar research, Miguez-Pacheco et al. [231] observed the in vitro behavior of ST-2 cells in RPMI medium exposed to extracts of 45S5 BG containing Nb_2_O_5_ (0–1 mol%) powders. The results showed that the higher tested concentration of 10 mg/ml was toxic to cells, while 1 and 0.1 mg/ml concentrations did not show a negative effect on cells. When compared to undoped 45S5 BG, different Nb contents did not show significant effects on cell viability at low concentrations (0.1 and 1 mg/ml). On the other hand, at lower concentrations, there was a significant release of VEGF from ST-2 cells, indicating the potential angiogenic effect of Nb-BG. Furthermore, in vivo studies [232–234] showed the osteoestimulative potential of Nb-doped bioactive glass for bone replacement. Figure 4 illustrates the subperiosteal bone region growth promoted by Nb-45S5 BG (46.1 SiO_2_–26.9 CaO–24.4 Na_2_O–1.3 P_2_O_5_–1.3 Nb_2_O_5_ mol.%) cylindrical rods after 28 days of implantation into a defect in rat calvaria with dimensions of 4 mm length and 2 mm diameter [232]. Similarly, Fig. 5 shows fully bone regeneration in a 5 mm rat calvarial defect after 8 weeks of implantation. In this study, a higher amount of Nb compared to the previous investigation was used (2.6 Nb_2_O_5_) [233]. Phosphate-based glasses-containing Nb have also been reported by Obata et al. [235, 236]. The biological properties of Nb-containing phosphate BGs (3 and 5 mol% Nb_2_O_5_ in the composition 60 CaO–30 P_2_O_5_–10 Na_2_O in mol%) demonstrated higher ALP activity for Nb-BGs compared to Nb-free phosphate BG as well as an influencing effect on differentiation and mineralization dependent on Nb concentration [236]. The incorporation of higher amounts of Nb_2_O_5_ (0–60 mol%) in phosphate glasses has also been investigated [237]. Lima et al. [219] studied in vivo the effect of 30 mol% Nb_2_O_5_ in the system P_2_O_5_–BaO–K_2_O after the implantation of granules in a rat model. After 3 and 9 weeks of implantation the authors reported blood vessel formation and no fibrous capsules around the granules.

#### Chromium (Cr)

Chromium is one of the essential elements in the human body. It has a biological role that influences the activity of insulin receptors [238]. Furthermore, chromium is one of the major trace elements regulating blood sugar and lipid levels in the body [239]. Recent reports indicate that an intake of 120 μg of chromium per day is sufficient for adults to preserve their health [240]. Toxic daily doses exceed 200 μg [240]. In bioactive glasses and bioceramics, chromium has shown promising effects by enhancing bioactivity, antibacterial activity, and degradation properties [241]. Krishnamacharyulu et al. [241] investigated a chromium-doped calcium borosilicate glass produced via the conventional melt-quenching method with composition 43 B_2_O_3_–5 SiO_2_–2 P_2_O_5_–20 Na_2_O–20 CaO (mol%). Varying concentrations of chromium oxide, ranging from 0 to 1 mol%, were incorporated in the BG. It was reported that the presence of Cr_2_O_3_ as a network modifier changed the structure of the glass by breaking the network bonds and causing the formation of non-bridging oxygen. Furthermore, the increment of Cr_2_O_3_ concentrations enhanced chromium ions transfer from tetrahedral chromates (CrO_4_^2–^) to octahedral chromates (CrO_6_), reducing the glass strength. The degradation rate of the glass in SBF increased for higher contents of Cr_2_O_3_ due to octahedral chromates positions. The substitution of Cr_2_O_3_ with CaO led to apatite formation in SBF solution after 28 days. Furthermore, the intensity of the XRD peak corresponding to HA increased as the Cr_2_O_3_ concentration increased. Hence, with an increase in the Cr_2_O_3_ content, the BG exhibited a superior bioactive behavior. The authors concluded that a high concentration of Cr_2_O_3_ (1 mol%) promoted greater BG degradation and in vitro bioactivity.

#### Molybdenum (Mo)

Molybdenum is a trace element required for several enzymes such as xanthine oxidoreductase, sulfite oxidase, and mitochondrial amidoxime reductase, being important for the metabolism of purines, sulfur-containing aminoacids, conversion of aldehides to acids, protein synthesis stimulation, and body growth [242–245]. In the human body, molybdenum is found primarily in the adrenal glands, bones, liver, and kidneys [246]. For biomedical applications, Mo-containing biomaterials are attracting attention due to their antibacterial and anticancerogenic properties [245, 247–254]. According to Ponta et al. [255], Mo-containing sol–gel derived SiO_2_–CaO–P_2_O_5_ BGs have potential for applications in bone tissue engineering by stimulating in vitro apatite formation in SBF solution after 10 days. MoO_3_ in the range of 3–10 mol% has been added and the influence of Mo on bioactivity and biocompatibility of the BGs was investigated. XRD patterns of Mo-doped BG calcined at 600 °C confirmed the presence of hydroxyapatite and calcium molybdate (CaMoO_4_) nanocrystals. Moreover, in vitro biological assays indicated that crystalline CaMoO_4_ phases led to improved biocompatibility by increasing adsorption of bovine serum albumin without hindering the formation of hydroxyapatite. The authors concluded that a 5 mol% MoO_3_ substitution resulted in enhanced bioactivity and biocompatibility [255]. Similarly, Dang et al. [256] investigated the influence of MoO_3_ on bioactive glass-ceramic (Mo-BGC) scaffolds for bone/interface applications using silicate glasses of composition 70 SiO_2_–25 CaO–5 P_2_O (mol%) with 2, 5, and 7.5 mol% of MoO_3_ substituted for CaO. The sol–gel method and 3D printing technology were used to fabricate the Mo-BGC scaffolds. The findings indicated that the addition of Mo to BGC scaffolds increased the compressive strength due to the formation of CaMoO_4_ phase during the calcination process of Mo-BGC powder at 800 °C. In vitro degradation in Tris-HCl buffer solution of Mo-BGC scaffold resulted in a lower weight loss compared to Mo-free scaffolds. Furthermore, the rate of release of Mo ions from the scaffolds was evaluated in Tris-HCl solution for up to 28 days. A gradual release was observed during the incubation time dependent on the Mo concentration. The release profiles did not show a final time point; therefore, after 28 days, Mo was still being released from all Mo-doped scaffolds. Moreover, in vitro cell experiments demonstrated that crushed scaffolds with 7.5 mol% of MoO_3_ at a concentration of 25 mg/ml increased chondrogenic differentiation of rabbit chondrocytes (RCs) and osteogenic differentiation of hBMSCs at days 3 and 7 when compared to Mo-free BGC. Interestingly, in vivo studies in rabbit osteochondral defects for 8 and 12 weeks showed that BGC scaffolds with 7.5 mol% MoO_3_ considerably enhanced cartilage/bone regeneration, demonstrating bi-lineage bioactivity [256]. Furthermore, Mo-containing phosphate-based glasses have also been investigated. For example, Lucacel et al. [257] reported the bioactivity and biocompatibility of melt-derived 48 P_2_O_5_–45 CaO–5 K_2_O–2 B_2_O_3_ (mol%) glass containing 1, 3, 5, or 7 mol% of MoO_3_. XRD analysis confirmed the amorphous structure of the BGs with different amounts of Mo. The capability of HA formation of the glasses was evaluated in SBF for 15 days. In contrast to the Mo-free glass, no HA crystalline phase on the surface of molybdenum-doped calcium phosphate-based glass was detected, this might be due to the formation of dominant Mo^5+^ ionic species on the surface inhibiting the migration of calcium and phosphate ions to the glass surface. Phosphate BGs containing molybdenum at 5 and 7 mol% exhibited biocompatibility and low toxicity to HaCaT cells [257]. In drug delivery applications, molybdenum oxide has been used to modify the network of phosphate glasses in order to control the degradation rate. El-Meliegy et al. [258] investigated melt-derived phosphate glasses (50 P_2_O_5_–30 CaO–20 Na_2_O, mol%) incorporating MoO_3_ (from 5 to 10 mol%) to tune glass dissolution and drug release. The dissolution rate in Tris-HCl buffer solution of phosphate glass-containing molybdenum was lower than the one of the reference phosphate glass without Mo due to the high valence of Mo oxide, which improves the bonding strength in the glass network. The surface of Mo-free phosphate glasses exhibited calcium phosphate deposits after 7 days of immersion in SBF; however, this was not the case for the Mo-doped glasses (5 and 10 mol%). Moreover, Mo-containing BGs have shown lower Vancomycin release rates than Mo-free phosphate glass, which the authors attributed to the hydrogen interactions between the hydroxyl and amino-functional groups in the drug and the hydrated P–O–H groups in the phosphate glass network [258].

#### Vanadium (V)

Vanadium is a trace element related to nutritional and biochemical functions in humans, animals, and plants [259]. Daily consumption of 10 mg of vanadium per kilogram of body mass has been reported to not have negative effects on human health [260]. Biological properties of V include the ability to stimulate insulin synthesis and mimic the effects of growth factors and biomarkers for bone-forming cell differentiation [259, 261]; therefore, vanadium has been considered in BGs in various studies [253, 262–265]. Vanadium-containing borate-based bioactive glass (13–93B3 with 0.15–3 wt.% V) scaffolds have been investigated for bone tissue engineering applications [266]. Vanadium was reported to act as a network modifier in the 13–93B3 glass system, leading to a faster degradation in SBF solution under static conditions by inhibiting tetrahedral BO_4_ units formation. Moreover, 3 wt.% V-substituted 13–93B3 scaffolds exhibited crystalline HA after 20 days of immersion in SBF [266]. Similarly, in another study, Marzouk et al. [267] reported the bioactivity of V-containing borate glass (57.5 B_2_O_3_–17 CaO–5.5 Na_2_O–11 K_2_O–4.5 MgO–4.5 P_2_O_5_ in wt.% with 0.5–1 wt.% V) after immersion in phosphate solution for 14 days. Furthermore, Deliormanli et al. [268] investigated in vivo the capacity of vanadium incorporated borate-based BG scaffolds for soft tissue applications using a mouse subcutaneous implantation set-up. After implantation for 4 weeks, fibrous connective tissue infiltrated inside V-containing scaffolds. As the concentration of vanadium increased to 3 wt.%, a reduction of tissue filtration was observed. In addition, V-containing scaffolds (3 wt.%) were reported to have a negative effect on angiogenesis by decreasing the vascularization area compared to V-free 13–93B3 BG scaffolds. Furthermore, according to a recent study, V-doped borate-based 13–93B3 BGs have also shown potential to be used in medical radiation applications and luminescence bioimaging [269, 270].

Li et al. [271] used the hydrothermal synthesis technique to dope MBG in the system SiO_2_–CaO–P_2_O_5_ with vanadium in various concentrations (0, 0.71, 2.78, and 6.67 mol%) with a triblock copolymer (P123) as the structure-directing agent. The aim of the study was to modify the morphology and mesostructure of V-doped MBG to optimize the glass dissolution and biological behavior. Vanadium concentration significantly influenced the morphology and mesostructure of V-doped MBG. The mesopore size, total pore volume, specific surface area, wall thickness, total micropore volume, and ordered mesostructure decreased significantly at increasing V content due to the presence of vanadate anions in solution, that could change the P123 micellization and self-assembly behavior by inducing salting-in and acidity-down effects, as well as three different forms of vanadium species located at the pore walls and/or the surface of the MBG. Clearly, the number of studies on V-containing BGs is very limited and, therefore, the potential biological benefits of V in conjunction with BGs should be further investigated in systematic studies, considering also different silicate glass compositions.

#### Manganese (Mn)

Mn is an essential trace element, which is required for the growth, development, and maintenance of healthy bones; a lack of this element in the pre-natal and early post-natal stages has been reported to cause skeletal abnormalities [272]. Bioactive glasses containing Mn have been investigated due to the properties provided by this ion, such as bioactivity, biocompatibility, and antibacterial effects [273–281]. Miola et al. [282] reported the incorporation of Mn in a melt-derived silicate BG (45 SiO_2_–3 P_2_O_5_–26 CaO–7 MgO–15 Na_2_O–4 K_2_O) substituting the molar ratio of MgO by MnO in the range of 0.25–0.5%. In vitro bioactivity tests in SBF revealed that Mn-doped BG showed HA formation on the surface after 28 days. Moreover, the effect of Mn-doped BG on human MG 63 cells was also evaluated, indicating that 0.25–0.5 mol% MnO did not show any toxic effect within 5 days of incubation. Furthermore, Mn^2+^ has been shown to promote osteogenic gene expression described by the enhancement in ALP activity, type I collagen, osteocalcin, bone morphogenetic proteins, and soluble intercellular adhesion molecule-1 (sICAM-1) in osteoblasts. Since Mn-doped BGs have been shown to stimulate cell proliferation, cellular differentiation, and bioactivity, they are promising materials for bone tissue regeneration. In a different approach, Cañaveral et al. [283] investigated Mn-doped 58S sol–gel-based BG in which CaO was replaced by MnO (3–5 mol%). After calcination at 700 °C, the presence of Mn^2+^ significantly influenced the structure of 58S BG. XRD analysis revealed the presence of crystalline phases such as Ca_3_Mn_2_Si_3_O_12_, CaSiO_3_-MnSiO_3_, and CaSiO_3_ in Mn-doped BG while Mn-free 58S BG exhibited an amorphous structure. However, the crystallization of the Mn-doped BG did not have a negative effect on bioactivity since the presence of Mn^2+^ increased apatite formation after 2 days in SBF comparable with bare 58S BG. Similarly, Barrioni et al. [284] doped 58S sol–gel BG with Mn^2+^ and evaluated the influence of the doping ion on the osteogenic cell differentiation capability and cytotoxicity of 58S BG. Interestingly, in contrast to the results previously described, XRD analysis indicated amorphous glasses with and without Mn^2+^ from 2.5 to 5 mol%. Furthermore, MTT assays confirmed that the dissolution products of Mn-doped glass (100–10,000 µg/ml) were not cytotoxic for osteoblast cells (for 72 h). Moreover, the antibacterial activity against *B. subtilis*, *P. aeruginosa*, and *S. aureus* of sol–gel Mn-doped BG (0–7 mol% MnO_2_) was demonstrated in other studies by Nawaz et al. [285]. Westhauser et al. [286, 287] reported the biological evaluation of sol–gel derived mesoporous bioactive glass nanoparticles (MBGNs) doped with 5 mol% MnO_2_. In vitro experiments using BMSCs demonstrated that MBGN with 5 mol% MnO_2_ enhanced osteogenic differentiation by upregulating ALP, osteocalcin, osteopontin, and collagen α1 at a concentration of 1 mg/ml, although lower cell viability was reported at the same tested concentration. In summary, MBGNs with 5 mol% MnO_2_ showed a significant cytotoxic effect at days 14 and 21. On the other hand, Mn containing MBGN at a concentration of 0.1 mg/ml increased cell viability from day 7 and did not show any cytotoxicity effect, demonstrating the dose-dependent effect of this material on cell behavior. Furthermore, phosphate-based BGs prepared via sol–gel synthesis (20 Na_2_O–15 CaO–5 B_2_O_3_–5 SiO_2_–55 P_2_O_5_) with 0–1 mol% of MnO_2_ have been reported by Bragiel et al. [288]. In vitro bioactivity in SBF showed apatite formation on the glass surface after 7 days. A larger radius of Mn^2+^ compared to Ca^2+^ led to a faster network degradation of Mn-doped glasses, leading to a faster apatite mineralization in SBF. No cell biology studies have been reported on such phosphate Mn-BGs.

#### Gold (Au)

Gold has been incorporated in BGs to explore the enhancement of features for drug delivery, wound healing, photothermal therapy, and bone regeneration [289–304]. Sol–gel BGs doped with gold nanoparticles (AuNPs) (60 SiO_2_–32 CaO–8 P_2_O_5_ mol% with 0–0.2 mol% Au_2_O) have been studied by Magyari et al. [305]. XRD patterns indicated Au crystalline phases, while no crystalline peaks were detected in the Au-free BGs. The presence of AuNPs in the BGs significantly affected the in vitro bioactivity and biocompatibility. AuNPs-doped BGs exhibited apatite layer formation after immersion in SBF for 7 days. The morphology of apatite-like structures on the BGs surface was shown to be dependent on the amount of AuNPs, resulting in both spherical and flower-like shapes (0.2 mol% Au_2_O). Furthermore, BGs with 0.15 and 0.2 mol% Au_2_O promoted the proliferation of human keratinocyte cells. Similarly, Grandi et al. [306] synthesized 58S BG doped with AuNPs (0.1 and 1 wt.%). Interestingly, the antibacterial properties against *S. aureus* of the reference 58S BG were enhanced by the presence of Au, while no enhancing effect was observed against *E. coli*.

#### Nickel (Ni)

Nickel has been incorporated in BGs to improve properties related to radiation attenuation and bone regeneration [307–312]. Vyas et al. [313–315] developed 45S5 BG and 45S5 BG-ceramic (BGC) doped with NiO at different concentrations ranging from 0.41 to 1.65 mol% via the melt-quenching route. Compared to Ni-free 45S5 BGCs, an increase in density and mechanical properties such as microhardness, compressive, and flexural strength was observed with increasing NiO concentration [313, 315]. The incorporation of Ni did not have an effect on the amorphous structure of 45S5 BG, as well as no additional crystalline phases were observed for the glass-ceramics with nickel, which exhibited crystalline species characteristic of sodium calcium silicate (Na_2_Ca_2_Si_3_O_9_ and Na_2_CaSi_3_O_8_). Furthermore, it was reported that the presence of Ni did not influence the bioactive behavior of all tested systems that showed apatite formation after 1 day of immersion in SBF [313, 315]. The cytotoxicity of Ni-doped 45S5 BGs to rabbit derived-osteoblast cells was directly tested. An MTT study revealed that Ni-45S5 BGCs (0–1.65 mol%) did not show cytotoxic behavior, resulting in higher cell proliferation at 0.82 NiO mol% [313].

#### Palladium (Pd)

In the biomedical field, palladium has been used in biosensors [316] and anti-cancer treatments [317, 318]. Wu et al. [319] investigated the addition of palladium in sol–gel-derived MBG for catalytic applications to oxidize benzyl alcohol and obtain benzaldehyde, a component that is widely used in the food industry and pharmaceutics. The authors reported that by increasing the amount of PdCl_2_ above 1.2%, the catalytic activity was reduced, while concentrations between 0.46 and 0.96% led to an efficient catalytic activity.

#### Tungsten (W)

Tungsten has been considered as non-carcinogenic and non-teratogenic, and it does not hold metabolic properties in animals and humans. In addition, under illumination, it exhibits high photocatalytic activity and antimicrobial properties [320]. Tungsten has gained interest to be incorporated in bioactive glasses due to the potential radiocontrast properties that can be transferred to the material, for example, to visualize the bone restoration process or as radiation shielding material [321, 322]. In this sense, Medkov et al. [320] developed sol–gel-derived BGs based on the 45S5 composition with WO_3_ ranging from 0 to 4 wt.%. At increasing amounts of WO_3_, microcrystals enriched with tungsten and sodium tungstate were detected and increased radiocontrast values from 1.2 to 5.6 mm Al, respectively, which are in the adequate range values for monitoring processes. Furthermore, Deliormanli et al. [321] investigated the properties of a composite made of the borate 13–93B3 bioactive glass (5.5 Na_2_O, 11.1 K_2_O, 4.6 MgO, 18.5 CaO, 3.7 P_2_O_5_, 56.6 B_2_O_3_ wt.%) and tungsten disulfide (0–4 WS_2_ wt.%) for diagnostic imaging and radiotherapy applications. In terms of structure, the addition of WS_2_ in the composites resulted in denser materials with the formation of tungsten trioxide phases and enhanced photon attenuation ability.

### Halogens

#### Chlorine (Cl)

One of the essential electrolytes in the human body is chloride. It assists in properly regulating body fluids and the maintenance of fluid balance inside, outside or between cells [323]. Cl has been incorporated in bioactive glasses for application as additives in toothpaste to help prevent tooth hypersensitivity and promote apatite formation [324–326]. Moreover, chloride has been used as an alternative to fluoride, which has been extensively used in dental applications to prevent caries; however, high content of fluoride in BGs can lead to crystalline calcium fluoride instead of fluorapatite, which might cause dental fluorosis in children [325, 327, 328]. Highly degradable BGs in the system SiO_2_–P_2_O_5_–CaO–CaCl_2_ (with CaCl_2_ in the range of 0–16.6 mol%) have been produced by Chen et al. [327] via the melting route. These glasses exhibited the formation of an apatite-like phase within 3 h of immersion in Tris buffer and an increasing degradation rate dependent on the amount of CaCl_2_. Similarly, mixing chloride and fluoride in a glass composition in the form of CaF_2_ and CaCl_2_ has also been considered by Chen et al. [329] by the processing of melt-derived BGs in the system SiO_2_–P_2_O_5_–CaO–CaF_2_/CaCl_2_, with CaF_2_ content ranging from 1.5 to 13.4 and CaCl_2_ from 2.6 to 21.5 (mol%). It was reported that in terms of structural properties, there was no great difference between the BGs. However, due to the difference in the size of fluoride and chloride ions, the crystallization tendency was lower for chloride-containing BGs compared to fluoride BGs. In comparison, a series incorporating both ions resulted in glasses with a stronger crystallization tendency. In terms of material properties, the addition of chloride ions could lead to BGs for applications in mineralizing dental toothpaste or resorbable bone substitutes, although there is still a lack of a comprehensive biological evaluation of such systems.

#### Iodine (I)

Iodine has been considered an essential element in the human body since it is involved in the production, activation, and metabolism of the thyroid hormone [330]. The ability of iodine ions to provide borate-based BGs antibacterial properties and promote neuron regeneration has been investigated [114, 331, 332]. Ottomeyer et al. [331] reported the antibacterial effect against different bacteria of 13–93B3 BG doped with 2 wt.% iodine and compared the effect of iodine with that of other dopants such as silver and gallium. The authors reported differences in the bacteria sensitivity with all glass formulations, explained by the distinct mechanisms of the dopant ions. Iodine showed a significant antibacterial effect against *V. natriegens*, *S. sonnei*, *S epidermis*, and a more negligible effect than undoped 13–93B3 BG against *E. coli MRSA* and *M. catarrhalis*. The biological impact of iodine-containing BGs has been studied in vitro by Thyparambil et al. [114] and Gupta et al. [332]. The addition of 0.1 wt.% of I in the 13–93B3 composition led to an increased proliferation and migration capacity of ASC cells, resulting in a beneficial approach to stimulate endogenous cells and to accelerate healing processes [114]. In contrast, 0.2 wt.% of NaI in 13–93B3 BGs had a significant negative effect on neuron survival and regrowth compared to other dopants such as Cu or Ga.

### Other elements

#### Germanium (Ge)

Germanium is a trace element present in plants, animals, and humans [333]. It has been considered for the treatment of cancer, arthritis, and senile osteoporosis due to the therapeutic attributes such as immune enhancement, oxygen enrichment, and heavy metal detoxification [334]. Germanium containing silicate BGs have been investigated for applications as bone filling materials [335, 336]. Mokhtari et al. [336] investigated the structural properties of 45S5 BGs containing Zn, Sr, and Ge ions (48 SiO_2_–6 CaO–8 SrO–36 ZnO–2 P_2_O_5_ with 6 and 12 mol% GeO_2_) to be used as injectable polyalkenoate cement glasses for applications in spinal orthopedic procedures. Amorphous Ge-BGs showed enhanced bioactive behavior compared to the reference glass after immersion in SBF for 4 days, demonstrating that the formation of apatite-like structures was dependent on the amount of GeO_2_. Furthermore, the nuclear radiation shielding behavior of Ge containing glasses has been studied by Saddeek et al. [337] using computational tools. Alkaline phosphate glasses in the system P_2_O_5_–Na_2_O–K_2_O–BaO–Al_2_O_3_–Sb_2_O_3_–La_2_O_3_–Nb_2_O_5_–Y_2_O_3_–Yb_2_O_3_ (with 0–84 Mol% GeO_2_) were evaluated in terms of the effect of GeO_2_ on the glass mass attenuation parameter and the effective atomic number. Such values resulted increasingly dependent on the amount of GeO_2_ and indicated the possible use of these materials for gamma shielding applications. In addition, there was a mechanical reinforcement effect with the incorporation of Ge, evidenced in the stronger glass network identified for higher concentrations of germanium oxide.

#### Bismuth (Bi)

Bismuth is a heavy metal ion that possesses antibacterial properties and has been widely used in pharmaceutical applications for the treatment of syphilis, gastrointestinal affections, cancer, and wound infections [338–340]. Average Bi consumption in humans is reported to be between 5 and 20 µg per day [341]. Bismuth-reinforced BGs have shown potential applications for radiation shielding and bone regeneration [63, 251, 342, 343]. Bismuth ferrite (BF) has been considered as an effective reinforcement agent in bioactive glasses for stimulating bone tissue formation and accelerating ALP activity [344]. Under the application of magnetic fields of 350 mT during 30 min per day, the in vitro bioactivity and bone mineralization of a BF-containing bioactive glass (BF-BG) facilitated bone like-apatite deposition in SBF after 21 days [344]. The addition of 2 wt.% BF to BG led to two-fold and three-fold greater ALP activity of MC3T3-E1 cells after 7 and 14 days, respectively, compared to the original glass composition (57 SiO_2_–10 Na_2_O–22 CaO–6 P_2_O_5_–2 TiO_2_–3 Bi_2_O_3_ in wt.%) [344]. Furthermore, Bi-doped phosphosilicate bioactive glasses (Bi-PBGs) have also shown photothermal effects when exposed to an 808 nm laser diode demonstrating the potential effect of killing bone tumor cells and enhancing hydroxyapatite mineralization in SBF solution [345]. This study reported cell viability higher than 80% for different cell lines, namely, mouse fibroblasts (L929), MC3T3-E1, rat osteosarcoma-derived cells (UMR106), and human osteosarcoma cells (U2OS) [345]. Prasad et al. [346] investigated in vitro cell proliferation of mouse fibroblast (NIH3T3) and antibacterial properties of Bi containing S53P4 BG against *E. coli*. After 11 days, the percentage of cell proliferation exposed to Bi containing S53P4 BG (1 and 2 wt.%) became higher compared to the non-doped S53P4 glass. In terms of antibacterial properties, 1, 2, 4, and 8 wt.% Bi_2_O_3_-containing S53P4 glass demonstrated antimicrobial effect against *E. coli* with glass powder concentrations of 100 mg/ml incubated at 37 °C for 1 and 2 h. In addition, bismuth oxide-doped 45S5 BG nanoparticles showed potential properties for applications as dental root canal sealers [347] and radio-opaque Bi-doped 45S5 BGs produced by pyrolysis of organic solutions have been proposed to control the process of bone regeneration [168].

#### Tin (Sn)

Tin is a trace micronutrient for living organisms reported to be in lower amounts beneficial for cancer treatment [348, 349]. A couple of studies have considered the incorporation of Sn into the structure of glasses for biomedical applications [350]. Recently, Alfadhli et al. [350] reported the gamma ray interaction parameters of glasses in the system PbCl_2_–SnCl_2_–P_2_O_5_ (with SnCl_2_ content from 40 to 60 mol%) for applications in nuclear medicine. The BG of composition 35 PbCl_2_–45 SnCl_2_–20 P_2_O_5_ exhibited the lowest free path, tenth-value layer, and half-value layer showing superior efficiency to absorb gamma rays.

#### Nitrogen (N)

Nitrogen has been reported to enhance the mechanical behavior, antibacterial effect, and the photon attenuation of BGs [351–354]. Bachar et al. [355, 356] studied the influence of nitrogen on the density, hardness, and elastic modulus of melt-derived BGs (55 SiO_2_–13.5 CaO–31.5 Na_2_O, mol.%) at various concentration of Si_3_N_4_ from 0 to 4 mol% [355] and 55 SiO_2_–8.5 CaO–31.5 Na_2_O–5 CaF_2_ mol% (with Si_3_N_4_ in concentrations of 0–4 mol%) [356]. The incorporated N atoms into the original tetrahedral SiO_4_ structure led to a stronger glass network. Consequently, properties such as density, hardness, glass transition temperature, and elastic modulus of N-doped BG significantly increased at higher N concentrations, while the bioactive behavior decreased [355, 357]. Similarly, bioactive oxynitride glasses (55 SiO_2_–13.5 CaO–29 Na_2_O–2.5 P_2_O_5_ mol%) with increasing concentration of Si_3_N_4_ (up to 4 mol%) were studied [357]. In addition to the previously mentioned mechanical properties and bioactivity, these BGs exhibited nontoxic behavior to epithelial cells (L132 cells) at glass powder concentrations of 25–400 mg/l. [357]. Moreover, Marin et al. [358] investigated in vitro the biological behavior of 45S5 BGs doped with Si_3_N_4_ (5 and 10 mol%). The results revealed that the incorporation of Si_3_N_4_ into 45S5 BG had a stimulatory effect on the proliferation of SaOS-2 cells and enhanced osteogenic expression for collagen, osteocalcin, and osteopontin [358].

#### Tellurium (Te)

Tellurium is a trace element found in the human body, mainly in bones (90%), muscles (3%), fat (3%), and liver (1.2%) [359]. Besides, Te has been used to enhance biocompatibility [360], bioactivity [361], and radiation shielding properties [362, 363] of materials for medical applications [364, 365]. Damrawi et al. [361] investigated the bioactivity of tellurite and silicate glass for bioactive implants and dental materials. In vitro bioactivity tests on tellurite glass (50 TeO_2_–26 Na_2_O–21 CaO–3 P_2_O_5_ mol%) and silicate glass (50 SiO_2_–26 Na_2_O–21 CaO–3 P_2_O_5_ mol%) demonstrated that TeO_2_ led to accelerated hydroxyapatite nucleation and crystallization compared to the silicate BG after being soaked in SBF for 5 days [361]. In another research, Miola et al. [366] investigated the effects of tellurium (0–5 mol%) on bioactivity and biological behavior of BGs in the melt-derived system SiO_2_–Na_2_O–CaO–P_2_O_5_ for infection and inflammatory response regulation and to improve bone tissue regeneration. In terms of structural information, Raman spectra of the BGs indicated that Te-incorporated BGs consist of TeO_4_ and TeO_3_ structural units. Furthermore, XRD analysis demonstrated that Te had no influence on the amorphous nature of the glasses. The addition of 1 mol% TeO_2_ resulted in the precipitation of HCA in SBF after 3 days, whereas 5 mol% Te-containing BG delayed the bioactive behavior in SBF considerably. Compared to Te-free BG, Te-containing glasses demonstrated significant antibacterial and antioxidant effects. Furthermore, the viability of hBMSCs was not negatively affected by the presence of Te in the BGs. Besides, due to tellurium’s capacity to prevent the generation of harmful oxygen and nitrogen active species, the metabolic activity of cells in contact with Te-BG under H_2_O_2_ stress was also evaluated, demonstrating the protecting effect of Te ions to cells. Antibacterial tests revealed that Te-containing glasses had a strong antibacterial effect, inhibiting biofilm formation of *S. aureus* and *S. epidermidis*. After 48 and 72 h inoculation, 5 mol% Te-doped BG had a significant effect on biofilm reduction compared to 1 mol% Te-containing BG [366].

#### Selenium (Se)

Selenium is an important element for humans in the form of selenocysteine, which is used in enzyme catalysis [367]. Se is particularly vital for the brain, since its lack could lead to irreversible brain damage [367]. In addition, it has been shown that Se intake might be used as a chemopreventive treatment in patients at high risk of pancreatic cancer [368]. Se-doped BGs have shown significant properties for radiation shielding and bone regeneration applications [369–373]. MBGs (80 SiO_2_–15 CaO–5 P_2_O_5_ in mol%) incorporating 5 mol% of selenium (Se-MBGs) have been shown to induce in vitro apatite-forming ability (after 1 days immersion in SBF) [374]. Moreover, Se-MBG was successfully used as a drug delivery system for bone tissue therapy. Thanks to the higher surface area (242 m^2^/g) compared to MBG without Se dopants (235 m^2^/g), certain oxygen voids and lattice defects caused by the replacement of Si^4+^ with Se^6+^ allowed Se-MBG to provide a high DOX-loading efficiency (50%) [374]. The hardness and in vitro biological behavior of selenium oxide-doped 45S5 BG (0.75–6 wt.% Se) have been investigated by Karakuzu-Ikizler et al. [375]. Se incorporation improved the Vickers hardness of the BG. Moreover, cell viability of up to 80% was observed in 45S5 BGs modified with 0.75, 1.5, 3, and 6 wt.% of SeO_2_ after 24 h and 7 days of incubation with SAOS-2 osteoblast-like cells using extracts concentration of 5 mg/ml [375]. Besides, compared to 45S5 BG, Se-doped 45S5 BG accelerated the mineralization process in vitro but presented lower APL activity [375]. Hu et al. [376] evaluated the cytotoxic effect of selenium doping in mesoporous bioactive glass nanospheres (60 SiO_2_–(36–*x*) CaO–4 P_2_O_5_–*x* SeO_2_ with *x* = 0, 1, 3, and 5 in mol%). MG 63 osteosarcoma and MC3T3-E1 preosteoblast cells were incubated for 48 h with Se-MBG supernatants. All the Se-MBG-containing nanospheres were significantly toxic for MG 63 cells. However, the Se^4+^ ion concentrations (0, 1, and 3 in mol%) in MBG nanospheres were nontoxic to MC3T3-E1 cells, while 5 mol% Se (5Se-MBG)-containing nanospheres were significantly toxic for MC3T3-E1 cells at concentrations higher than 20 µg/ml [376]. 3Se-MBG and 5Se-MBG nanospheres showed a significant apoptosis effect on MG 63 cells compared to the control. The Se-free MBG and other Se-MBG nanospheres showed no obvious ability to induce apoptosis [376]. Alternatively, DOX was successfully loaded into Se-MBG nanospheres to improve the viability of MG 63 cells resulting in slightly higher viability than the positive control (free DOX). Moreover, selenium has shown antibacterial effects. This antibacterial activity has been demonstrated incorporating Se-doped borosilicate glass nanoparticles (80 SiO_2_–18 B_2_O_3_–2 SeO_2_ mol%) in alginate-agarose polymeric blends designed for wound healing applications. The presence of Se-doped borosilicate glass in the polymer showed a significant antibacterial effect against *S. aureus* and *Candida albicans* compared to only alginate-agarose blend [377].

## Discussion

Bioactive glasses are attracting considerable attention for regenerative medicine and tissue engineering applications due to their excellent features in terms of bioactivity, biodegradability [378], osteogenesis [51], angiogenesis [11], antibacterial [95], anti-inflammatory [379], and immunomodulatory effects [380, 381]. The field of ion releasing BGs for biomedical applications has been growing in the last 20 years, and several comprehensive reviews on different aspects of ion releasing BGs for biomedical use are available [39–41, 49, 53, 54]. More recently, BG compositions incorporating exotic (“exotic” in the sense that such ions are not obviously linked to a biomedical use due to a possible biological activity) or less-common ions have started to be investigated. Numerous studies have shown the use of those ions in silicate-based systems; however, progress has also been made in borate and phosphate glasses. The potential application of BGs relies on their synthesis method, structure, and composition. The incorporation of various therapeutic elements into bioactive glasses has the aim to enhance not only the physical and mechanical properties of the material but, specially, to impart additional features such as bioactivity, biodegradability, osteogenesis, angiogenesis, and antibacterial properties [41]. As can be seen in Table 1, BGs doped with “less-common” ions, namely, rare earth elements and other less obvious ions for biomedical use, have been produced by both the conventional melting-quench and sol–gel methods and have been shaped or processed to achieve several morphologies. Mesoporous BGs produced by sol–gel have shown outstanding characteristics to be used in drug delivery applications [53]. Furthermore, BGs are used for the production of 3D-porous scaffolds fulfilling certain properties such as adequate porosity, mechanical stability, pore interconnectivity, and biocompatibility to facilitate nutrient supply and they can act as suitable signaling templates for bone and soft tissue regeneration [51]. These materials have also been applied as particles or granules to be directly implanted inside a defect [382]. Moreover, BG fibers can exhibit well-ordered structures (e.g., parallel fibers), leading to higher mechanical properties and high bioactivity in SBF [383] as well as suitable properties for drug delivery [59].

The ability of BGs to promote the formation of hydroxyapatite on their surfaces is important as this determines their tissue bonding capability, particularly to hard tissue. The in vitro apatite formation can partially predict the bone formation capacity of doped bioactive glasses. The tunability and control of ion release overtime during the dissolution of BGs have been increasingly investigated to develop bioactive glasses capable of supporting (hard and soft) tissue regeneration by tailored release of biologically active ions. The formation of new bone promoted by BGs can be linked to their chemical durability and dissolution rate in biological fluids. For example, BGs containing less-common metal ions have gained special attention due to the positive effect of such ions on the material (BG) bioactive character. For example, the substitution of Eu, Sm, Y, La, Rb, Bi, Se, Zr, and Ta has been shown to lower chemical durability, which favors apatite formation when the BGs are immersed in SBF solution. On the other hand, glass dissolution decreased in the case of Gd-doped bioactive silicate glass in the system SiO_2_–Na_2_O–CaO–P_2_O_5_ with 2.5 wt.% Gd_2_O_3_. Still, Gd-doped BGs exhibited high bioactivity after soaking in SBF, indicating that the slow glass dissolution of that particular BG composition had no negative effect on bioactivity in terms of hydroxyapatite formation [85].

In vitro degradation studies in SBF or Tris-HCl buffer solutions have shown that the incorporation of Ba^2+^, Cr^3+^ (in silicate BGs), and V^5+^ (in borate BGs) enhanced the degradation rate of BGs, resulting in superior bioactive behavior. Moreover, increasing concentrations of oxides of Ba (0–10 mol% [154]), Cr (0–1 mol% [241]), and V (0.15–3 wt.% [266]) boosted the crystallization of hydroxyapatite on BG surfaces. Furthermore, there is no agreement in the literature on the effect of Mo oxide on the bioactivity of BGs. Ponta et al. [255] reported that the incorporation of Mo oxide (5 mol%) resulted in a silicate-based BG exhibiting bioactive behavior after 10 days of immersion in SBF; however, Lucacel et al. [257] and El-Meliegy et al. [258] reported that BGs containing Mo^6+^ (1–10 mol% [257, 258]) did not induce hydroxyapatite formation after 15 days in SBF. This result was explained by the presence of dominant Mo^5+^ ionic species on the surface inhibiting the migration of Ca^2+^ and PO_4_^3−^ groups to the glass surface [257]. Moreover, the addition of Ba to borosilicate glasses might improve the radiation shielding ability of the materials, described by the fact that increasing amount of BaO in the glass system produces an increase of the glass density resulting in enhanced resistance to gamma radiation [160].

Materials intended to be implanted in the body or in contact with open wounds must exhibit a number of properties linked to their biocompatible characteristics. Toxic effects can cause harm to the host tissue and should be prevented. The addition of less-common ions, which are not obviously considered for their cell biology activity, must include an assessment of biotolerance as function of concentration. As a result, the possible toxic effects of incorporating different ions in BGs require careful investigation both in vitro and in vivo. In this context, the influence of doping BGs with Eu, Gd, La, Bi, Se, Zr, and Nb on living cells, such as mouse fibroblasts L929 [384], macrophages (RAW 264.7) [69], osteoblasts (MC3T3-E1) [60], BHK fibroblasts [118], rat osteosarcoma-derived (UMR106) [345], and human osteosarcoma (U2OS) cells [201], has been investigated and always an ion dose-dependent response has been found. Results have shown that dissolution products of BGs containing the mentioned ions are nontoxic at low concentrations. Similarly, bioactive glasses with doping ions such as Ba^2+^, Mo^6+^, and Te^4+^ did not show any cytotoxicity effect on different cell lines, for example, on glioblastoma cells and granulocytic 466 cells (at 1.35 mol% Ba-doped BG) [9], L929 mouse fibroblast (0–10 mol% Ba [154]), human bone marrow-derived stem cells (0–5 mol% Te [366]), as well as RCs and human bone marrow-derived stem cells (7.5 mol% Mo [256]). Even though progress has been made on investigating cytotoxicity, to the authors’ knowledge, no specific studies on the cytotoxicity of Sm-, Y-, Cr-, V-, and Rb-containing bioactive glasses have been reported so far and this is an important aspect that should be investigated in more detail to take advantage of the therapeutic properties that these ions could provide for tissue regeneration.

A challenge for tissue engineering is to devise an effective approach to use biomaterials that are not only suitable in terms of mechanical stability (according to the host tissue) but also promote the relevant healing and regenerative processes including angiogenesis as a key requirement for both soft and bone tissue engineering. Adding “less-common” ions to BGs is an approach that is becoming highly considered in parallel to the use of the more “standard” ions such as Sr, Cu, Zn, Ag, Mg, and Co. For example, the use of Eu, La, and Rb in silicate-based bioactive glasses led to improved osteogenic and angiogenic responses in mouse bone marrow stromal cells, hBMSCs, and endothelial-like cells (HUVECs). Furthermore, finding the right concentration of doping ions is important to gain information on their toxic levels and also to determine the minimum amount necessary to provide a therapeutic effect. Therefore, in vitro studies have been carried out using different ionic concentrations. For example, Eu^3+^ (in the range of 6.25–25 mg/ml) [70], La^3+^ (50 mg/ml) [117], and Rb^+^ (100 mg/ml) [141] incorporated in different BGs have been shown to activate the Wnt/β-catenin, and HIF-1α signaling pathways to upregulate the secretion of osteogenic genes (RUNX2, ALP, OPN, OSX, and BSP and COL I) as well as the promotion of angiogenic growth factors (b-FGF, VEGF, PDGF and, CD31, PDGFRα/β, VEGFR1/2, and MMP9). High concentrations of doping ions, on the other hand, resulted in being harmful to cells [70]. Other elements, such as Gd [89, 90], Zr [198], and Nb [230], have been shown to enhance osteoblast activity when tested in vitro with rBMSCs, MC3T3-E1, and BMSCs cells, respectively.

Photoluminescence features of rare earth ions have been investigated in MBG fibers doped with europium and samarium. These rare earth ion-doped BGs have the potential to be used in bioimaging, for instance, for the in vivo monitoring of new bone growth in bone defects [70] and in applications where monitoring the material degradation is desired [96] or as drug delivery carriers [56, 58]. Furthermore, the inhibitory activity of Eu-doped MBGs on the expression of pro-inflammatory factors such as IL-18, IL-6, IL-1, OSM MyD88, Ticam1, TNF-α, and Ticom2 has also been investigated [69, 379]. Clearly, such ions offer an interesting combination of functional properties and biological effects, which cannot be obtained by the classical doping ions.

Infection is a major cause of implant failure, being bacterial adhesion and biofilm formation the main causes of infection [385]. Bioactive glasses doped with metal cations such as rubidium [141], selenium [377], and zirconium [183] have been shown to impart high antibacterial activity against *S. aureus*, *E. coli*, and *P. aeruginosa*. Likewise, tellurium has been described as a doping ion that promotes antibacterial effects and antioxidant effects on BGs. Antibacterial properties against *S. aureus* and *S. epidermidis* have been reported for BGs with high TeO_2_ concentrations (5 mol%) [361].

Furthermore, Rb-doped mesoporous glass scaffolds have been developed as promising templates for drug loading [141]. Indeed, the long-term consequences of bacterial resistance to antibiotics give future perspectives for the development of new antibiotic-free materials for use in medicine. Bioactive glasses have great potential in this field, especially when antibacterial ions are incorporated in the right amount and can be released in a controlled manner representing an alternative antibacterial technology. In this context, the dual release of antibiotics and antibacterial ions from MBGs is a powerful emerging approach, as recently discussed [386], exploiting synergies that can emerge by the simultaneous release of ions and biomolecules.

Since bioactive glasses have shown drawbacks in terms of mechanical properties and fracture resistance, research focusing on alternatives to improve such properties is being increasingly carried out. The mechanical strength of BGs can be tailored by adjusting the chemical composition and by inducing crystallization [387]. As a result, several ions, including selenium and zirconium, have effectively been incorporated into bioactive glasses to enhance their mechanical properties. The development of new crystalline phases such as ZrSiO, ZrSiO_4_, Zr_2_O(PO_4_), and Ca(ZrO_3_) has been shown to increase the compressive strength of Zr-containing BGs. However, the controlled crystallization of BGs incorporating less-common ions as an strategy to obtain better mechanical properties has not been extensively exploited so far.

Based on the results reported in the literature, which have been summarized and discussed in this review, it can be stated that there is still a lack of studies evaluating the long-term performance of BGs incorporating less-common ions, especially with an assessment of their biological behavior in vivo, including long-term studies to assess possible delayed cytotoxic effects of such ions. In addition, more studies need to be carried out considering the applications of ion-doped BGs in the production of 3D constructs and scaffolds since most of the reported studies have considered BGs in particulate form. In comparison to the much higher amount of data on BGs containing “classical” ions such as Cu, Sr, B, Li, Mg, K, Co, studies on BGs incorporating “less-common” ions discussed in this review are scarce; however, the field is highly promising and is expanding, with new research continuously generating data to complete our understanding about the properties and applications of such BGs.

## Conclusions

According to this literature review, research is increasingly focusing on improving the properties of bioactive glasses by doping them with less common dopants, including rare-earth elements. The addition of these dopants alters the bioactive glass properties imparting novel functionalities and induces specific biological effects. The use of rare earth elements in bioactive glasses also expands their medical applications, considering the achieved therapeutic effects combined with functional properties (e.g., for imaging applications). In this paper, we have reviewed and discussed current knowledge on the effects of less-common ions on the properties of bioactive glasses, as summarized in Table 3. We anticipate further expansion of research on this particular class of BGs and propose this review as a timely addition to the literature for the benefit of those researchers entering the field.

**Table 3:** Effects “less-common” ions incorporated in bioactive glasses

Ion	Effects	Ref.
Barium	Apatite-forming bioactivity	[9]
	Biocompatible behavior	[9]
	Anti-inflammatory properties	[9]
	Gamma radiation properties	[160]
	Increases density transmission and optical bandgap	[160]
Bismuth	Apatite-forming bioactivity	[344]
	Increases the expression of ALP	[344]
	Biocompatible behavior	[345]
	Antibacterial property against gram-negative bacteria	[346]
Chlorine	Increases apatite-forming bioactivity	[324–327]
	Decreases glass durability	[327]
Chromium	Decreases glass durability	[241]
	Bioactive behavior	[241]
Dysprosium	Controlling drug release	[133]
Europium	Photoluminescence properties	[58, 59, 69, 70]
	Controlling drug release	[57–59]
	Promoting osteogenesis and angiogenesis potential	[69]
	Increases cell viability	[57]
	Increases apatite-forming bioactivity	[60]
	Increases the expression of ALP, COL1, and Runx2 genes and promoted osteogenic differentiation of BMSCs	[70]
	Decreases glass durability	[70]
Gadolinium or Ytterbium or Thulium	Increases glass durability	[85]
	Biocompatible behavior	[85]
	Promoting proliferation and differentiation of rBMSCs cells and human exfoliated deciduous teeth (SHED)	[85, 89, 90]
	Promoting newly formed bone and collagen deposition in rats, calvarial defect model, after 12 weeks post surgery	[90]
	Decreases the average particle size	[91]
	Photoluminescence properties	[91]
Germanium	Increases apatite-forming bioactivity	[336]
	Nuclear radiation shielding behaviors	[337]
	Increase bulk modulus and Young’s modulus	[337]
Gold	Antibacterial property against gram-positive and gram-negative bacteria	[306]
	Apatite-forming bioactivity	[305]
Holmium	Promoting preosteoblast cell proliferation	[78]
	Biocompatible behavior	[78]
	Bioactive behavior	[78, 79]
Iodine	Increases proliferation and migration capacity of ASC cells	[114]
	Antibacterial properties against *V. natriegens*, *S. sonnei*, *S epidermis*, *E. coli MRSA*, and *M. catarrhalis*	[331]
	Negative effect on neuron survival and regrowth	[332]
Lanthanum	Decreases polymerizing silica network	[124]
	Increases compressive strength	[124]
Manganese	Increases apatite-forming bioactivity	[282]
	Promoting osteogenic properties in vitro	[282]
	Biocompatible behavior	[284]
	Antibacterial properties against gram-positive and gram-negative bacteria	[285]
Molybdenum	Increases mechanical strength	[255]
	Biocompatible behavior	[255]
	Decreases glass durability	[256]
	Controlling drug release	[257]
Nickel	Increase density, microhardness compressive strength, and flexural strength	[313–315]
	Biocompatible behavior	[314]
Niobium	Increases apatite-forming bioactivity	[230]
	Biocompatible behavior	[229, 230, 233]
	Increases chemical durability	[229]
	Increases Vickers microhardness and compressive strength	[229]
	Promoting osteogenic and osteostimulative properties	[230, 232, 233]
Nitrogen	Increase density, hardness, glass transition temperature, and elastic modulus	[355–357, 394]
	Biocompatible behavior	[357]
	Increase osteogenic expression for collagen, osteocalcin, and osteopontin	[358]
Palladium	High catalytic activity on benzyl alcohol oxidation	[319]
Rubidium	Biocompatible behavior	[118, 123]
	Increases apatite-forming bioactivity	[346]
	Promoting angiogenesis and osteogenesis of hBMSCs	[118]
	Antibacterial property against gram-positive and gram-negative bacteria	[140, 346]
	Increases density and tensile strength	[141, 142]
	Antibacterial properties against gram-negative bacteria	[346, 395]
Samarium	Increases density, Young’s modulus, bulk modulus, and shear modulus	[97]
	Increases apatite-forming bioactivity	[97, 98]
	Photoluminescence properties	[93]
	Controlling drug release	[98]
Selenium	Increases apatite-forming bioactivity	[375, 376, 390]
	Controlling drug release	[376]
	Increases Vickers microhardness	[375]
	Biocompatible behavior	[375]
Tantalum	Increases apatite-forming bioactivity	[173]
	Antibacterial properties against gram-positive and gram-negative bacteria	[173]
	Biocompatible behavior	[162]
	Promoting hemostasis	[162]
Tellurium	Apatite-forming bioactivity	[366]
	Antibacterial properties against gram-positive and gram-negative bacteria	[366]
	Antioxidant properties	[366]
Terbium and Erbium	Biocompatible behavior	[59, 130]
	Photoluminescence properties	[59, 396]
	Increases apatite-forming bioactivity	[59, 130, 396]
Tin	High gamma rays efficiency	[350]
Tungsten	Increases radiocontrast values	[320]
	Increases density, Vickers microhardness, and compressive strength	[321]
	Enhancing photon attenuation ability	[321]
Vanadium	Decreases glass durability	[266]
	Apatite-forming bioactivity	[266]
	Photoluminescence properties	[269]
	Gamma radiation properties	[270]
Yttrium	Increases glass durability	[112, 113]
	Increase apatite-forming bioactivity	[105]
	Promoting proliferation and migration of adipose stem cells (ASCs)	[114]
Zirconium	Increases apatite-forming bioactivity	[175, 197]
	Decreases glass durability	[197]
	Decreases polymerizing silica networks	[197]
	Increases density, Vickers microhardness, compressive strength, and fracture toughness	[175, 183, 197, 198]
	Antibacterial properties against gram-positive and gram-negative bacteria	[175, 198]
	Biocompatible behavior	[175]
	Promoting proliferation and activity of osteoblast-like cells	[198]
